# Diversification of Campylobacter jejuni Flagellar C-Ring Composition Impacts Its Structure and Function in Motility, Flagellar Assembly, and Cellular Processes

**DOI:** 10.1128/mBio.02286-19

**Published:** 2020-01-07

**Authors:** Louie D. Henderson, Teige R. S. Matthews-Palmer, Connor J. Gulbronson, Deborah A. Ribardo, Morgan Beeby, David R. Hendrixson

**Affiliations:** a Department of Life Sciences, Imperial College London, London, United Kingdom; b Department of Microbiology, University of Texas Southwestern Medical Center, Dallas, Texas, USA; University of Hawaii at Manoa

**Keywords:** C ring, FlhG, FliI, FliN, FliY, flagellar motor, polar flagella, type III secretion

## Abstract

Bacterial flagella are reversible rotary motors that rotate external filaments for bacterial propulsion. Some flagellar motors have diversified by recruiting additional components that influence torque and rotation, but little is known about the possible diversification and evolution of core motor components. The mechanistic core of flagella is the cytoplasmic C ring, which functions as a rotor, directional switch, and assembly platform for the flagellar type III secretion system (fT3SS) ATPase. The C ring is composed of a ring of FliG proteins and a helical ring of surface presentation of antigen (SPOA) domains from the switch proteins FliM and one of two usually mutually exclusive paralogs, FliN or FliY. We investigated the composition, architecture, and function of the C ring of Campylobacter jejuni, which encodes FliG, FliM, and both FliY and FliN by a variety of interrogative approaches. We discovered a diversified C. jejuni C ring containing FliG, FliM, and both FliY, which functions as a classical FliN-like protein for flagellar assembly, and FliN, which has neofunctionalized into a structural role. Specific protein interactions drive the formation of a more complex heterooligomeric C. jejuni C-ring structure. We discovered that this complex C ring has additional cellular functions in polarly localizing FlhG for numerical regulation of flagellar biogenesis and spatial regulation of division. Furthermore, mutation of the C. jejuni C ring revealed a T3SS that was less dependent on its ATPase complex for assembly than were other systems. Our results highlight considerable evolved flagellar diversity that impacts motor output, biogenesis, and cellular processes in different species.

## Abstract (precis)

The conserved core of bacterial flagellar motors reflects a shared evolutionary history that preserves the mechanisms essential for flagellar assembly, rotation, and directional switching. In this work, we describe an expanded and diversified set of core components in the Campylobacter jejuni flagellar C ring, the mechanistic core of the motor. Our work provides insight into how usually conserved core components may have diversified by gene duplication, enabling a division of labor of the ancestral protein between the two new proteins, acquisition of new roles in flagellar assembly and motility, and expansion of the function of the flagellum beyond motility, including spatial regulation of cell division and numerical control of flagellar biogenesis in C. jejuni. Our results highlight that relatively small changes, such as gene duplications, can have substantial ramifications on the cellular roles of a molecular machine.

## INTRODUCTION

The bacterial flagellar motor harnesses proton flux to generate torque (1). Proton flux through a ring of stator complexes exerts torque upon a rotor (2–4); torque is transmitted through a rigid periplasmic rod and flexible surface hook to an extracellular flagellar filament that coils as a helical propeller for propulsion (1). This rotor and a switch component that controls the direction of motor rotation together form a cytoplasmic ring, the C ring (5). Torque is generated by the action of conformational changes in the stator complexes upon the C ring (2, 6–13), whereas switching of motor rotation from counterclockwise to clockwise is driven by binding of the response regulator CheY, whose activity is linked to sensing by chemosensory receptors (14). The C ring is also required for efficient flagellar biogenesis, as it is necessary for full assembly of the flagellar type III secretion system (fT3SS), which secretes extracytoplasmic axial components of the flagellar motor (15–21). Understanding how the C ring contributes to these functions is crucial to understanding how flagellar motors function and have evolved.

In the model peritrichous flagella of Escherichia coli and Salmonella species, the C ring is composed of FliG, FliM, and FliN (22–26). FliG oligomerizes into a ring that forms the upper rim of the C ring (27, 28). FliG docks the C ring to the cytoplasmic face of the transmembrane MS ring, composed of FliF, by cofolding of the C- and N-terminal domains of FliF and FliG, respectively (23, 29, 30). FliM and FliN form the switch complex beneath FliG (31, 32). The middle domain of FliM contains a CheC-like domain that forms a continuous belt in the middle of the C ring (33, 34). CheY, the response regulator of the chemotaxis signal transduction system, binds a conserved N-terminal EIDAL motif in FliM to influence clockwise or counterclockwise motor rotation for chemotaxis (16, 35–39). Structural studies suggest that homologous surface presentation of antigen (SPOA) domains in the C terminus of FliM and FliN form a continuous spiral through FliM-FliN_3_ protomers that compose the lower rim of the C ring (31, 40). This region of the C ring is also an assembly platform for integration of an ATPase complex into the fT3SS. Spoke-like FliH bridges anchor a FliI_6_-FliJ complex beneath the fT3SS inner membrane export apparatus to FliM-FliN_3_ (9, 41–43), where the central stalk protein FliJ can interact with the cytoplasmic ring of FlhA (44, 45). This FliI_6_-FliJ complex facilitates efficient secretion by the export gate by activating the fT3SS export apparatus, although its mode of action is not completely understood (46–50).

Flagellar motor C rings do not all follow this compositional blueprint. For example, Bacillus subtilis and Thermotoga maritima encode FliY in place of FliN (51–53). The domain organization of FliY is similar to that of FliM, with a phosphatase domain of the CheC/CheX/CheY family and a SPOA domain (53, 54). While FliN and FliY are mutually exclusive in most bacteria, Leptospira species and *Epsilonproteobacteria*, including Campylobacter and Helicobacter species, produce both FliN and FliY, although FliY in these bacteria lacks active sites for phosphatase activity (55, 56). An initial study in Helicobacter pylori indicated that FliN and FliY are required for full flagellation and motility (56, 57). Structural and interaction studies demonstrated that FliY interacts with FliM and FliN separately to assemble a three-protein complex (57). Furthermore, H. pylori FliY-FliN and FliY-FliM heterodimers interact with FliH *in vitro*, but how these proteins contribute to C-ring composition and architecture in H. pylori remains unknown (57). The presence of both FliY and FliN SPOA-containing proteins in *Epsilonproteobacteria* prompts questions regarding the function and location of these proteins, how have they diverged relative to an ancestral FliN-like protein, and what selective benefits drove retention of two FliN/FliY-like proteins.

The additional complexity of the epsilonproteobacterial C ring coincides with additional functions, although how complexity and function are related is unclear. The Campylobacter jejuni motor contains a wider C ring that generates higher torque than do many other species, facilitating motility in viscous environments by enabling interaction with a correspondingly wider ring of more stator complexes, which are positioned by large periplasmic disk structures (7–9, 58). The C. jejuni C ring also has functions unrelated to motility. We previously demonstrated that C. jejuni *fliM* and *fliN* mutants have defects in spatial regulation of septal Z ring formation to result in nonviable minicells due to cell division at poles rather than symmetrically at the midpoint (59). Many bacteria prevent FtsZ from forming Z rings at polar regions using a Min system (60). However, C. jejuni lacks a conventional Min system, instead using FlhG, a homolog of MinD (59). How the C. jejuni C ring impacts FlhG activity to prevent division at polar regions is unknown. FlhG also regulates how many flagella are assembled in polar flagellates, including C. jejuni (59, 61), and likely functions with the FlhF GTPase in many polar flagellates to control monotrichous, amphitrichous, or lophotrichous flagellation patterns (61–65). One hypothesis on how these proteins function involves FlhF toggling between ON and OFF states upon the hydrolysis of bound GTP, which is stimulated by FlhG (65). In this model, FlhF in its GTP-bound ON state initiates an undetermined early step in flagellar biogenesis (62, 64). After the production of a polar flagellum, FlhG stimulates FlhF GTPase activity to produce a GDP-bound OFF state, repressing FlhF activity and preventing assembly of more than a single polar flagellum.

Considering the potentially enhanced protein composition of flagellar C rings and diverse functions of the C ring in C. jejuni cell biology, we sought to determine the composition and architecture of the C. jejuni flagellar C ring and its contributions to flagellar biogenesis, torque generation, motility, fT3SS assembly, and cell division. To this end, we used a variety of genetic, *in vivo* protein interaction, functional, immunofluorescent microscopy, and *in situ* electron cryo-tomographic structural analyses to better characterize the C. jejuni C ring. Our results suggest that duplication (or possibly acquisition) of an ancestral FliN-like protein enabled the evolution of distinct roles in the C. jejuni C ring. This diversification of two FliN-like paralogs bestowed functional adaptations that may have coevolved with the widening of the C. jejuni C ring and facilitated the integration of regulatory mechanisms for polar flagellar assembly and the prevention of division at poles for accurate symmetrical division.

## RESULTS

### Evolutionary relatedness and domain structure of flagellar C-ring proteins across bacterial species.

**FIG 1:**
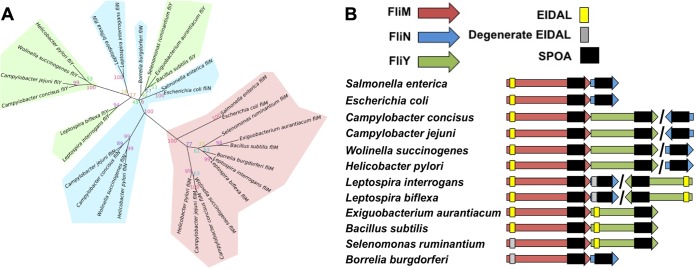
Phylogenetic analysis of the SPOA domain-containing family of flagellar proteins. (A) Unrooted phylogenetic tree of aligned sequences of FliM, FliN, and FliY from 12 bacteria from the spirochetes, *Firmicutes*, *Epsilonproteobacteria*, and *Gammaproteobacteria*. Confidence values were generated through 1,000 bootstrap repeats. Branches are shaded red for FliM, green for FliY, and blue for FliN. (B) Genomic organization and conserved encoded domains of *fliM*, *fliN*, and *fliY*. The SPOA domain (Pfam domain pf01052) is shown in black, and the presence of true (yellow) and potential degenerate (gray) EIDAL motifs is indicated. Adjacent genes indicate adjacent genomic locations, whereas slashes indicate distant locations.

To further the understanding of the roles of the C. jejuni FliM, FliN, and FliY proteins, we first estimated their phylogenies and studied their operon neighborhoods. Our maximum likelihood phylogeny of the common SPOA domain showed segregation of FliM from FliN and FliY (Fig. 1A), together with conservation of the EIDAL motif in most FliM sequences, which is separate from their SPOA domains. Full or degenerate EIDAL motifs were also sporadically present in FliN and FliY. Unexpectedly, however, FliN and FliY did not separate into discrete clades. Instead, distinct FliN and FliY clades were found in different bacterial lineages, such as the *Epsilonproteobacteria* and some spirochetes, suggesting independent ancestral duplication or horizontal acquisition of a second ancestral FliN-like protein allele in these distinct lineages, leading to multiple distinct origins of contemporary FliN and FliY. Indeed, our phylogeny suggests that the spirochete FliN is more closely related to the epsilonproteobacterial FliY than to the epsilonproteobacterial FliN, and vice versa, indicating that the epsilonproteobacterial FliN and FliY do not evolutionarily (or functionally) correspond to annotated FliN and FliY proteins in other species.

Consistent with this, operon analysis also showed differing patterns of *fliN* and *fliY* colocation with *fliM* (Fig. 1B). In peritrichous flagellates such as E. coli and *Salmonella* species, *fliM* and *fliN* are usually organized together within an operon, but in C. jejuni, *fliY* is immediately downstream of *fliM*, while *fliN* is located elsewhere. Our results suggest that presence of a CheC domain does not correspond to a single distinct FliY family. Rather, diverse species have evolved multiple SPOA domain FliN homologs, with some that incorporate a CheC domain, and some that do not (see Fig. S1 in the supplemental material), with one likely being a primary structural component. In peritrichous motors, FliN is the primary SPOA structural component, whereas FliY is the primary structural component in C. jejuni, with FliN presumably functioning in another role. The function of the SPOA domain is not correlated with the presence or absence of a CheC domain. Indeed, our results are not consistent with C. jejuni FliN being any more closely related to peritrichous FliN than with C. jejuni FliY.

10.1128/mBio.02286-19.1 FIG S1 Protein alignments of FliM, FliN, and FliY. Multiple-sequence alignments (MSA) generated using MAFFT and filtered using the T-coffee transitive consistency score. Sourced from 12 bacterial species from the spirochetes, *Firmicutes*, *Epsilonproteobacteria*, and *Gammaproteobacteria*. The SPOA domains of FliM, FliY, and FliN are indicated in the red box. Download FIG S1, TIF file, 1.9 MB. Copyright © 2019 Henderson et al. 2019 Henderson et al. This content is distributed under the terms of the Creative Commons Attribution 4.0 International license.

### Requirements of C. jejuni C-ring components for flagellar motility.

**FIG 2:**
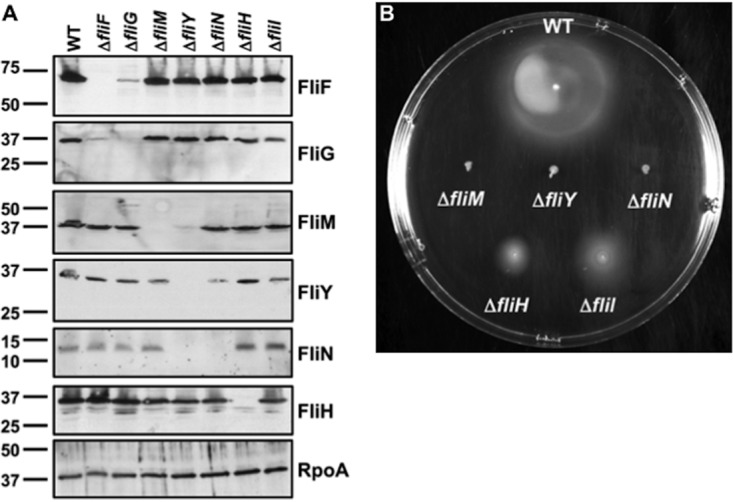
Flagellar protein stability and motility phenotypes of C. jejuni MS and C-ring mutants. (A) Immunoblot analysis of MS and C-ring protein levels in whole-cell lysates of WT C. jejuni and isogenic mutant strains. Specific antiserum to each MS and C-ring protein was used to detect each protein. The detection of RpoA served as a control to ensure equal loading of proteins across strains. (B) Motility phenotypes of WT C. jejuni and isogenic mutant strains. Motility was analyzed after 30 h of incubation at 37°C under microaerobic conditions in MH motility agar (0.4%).

For an initial assessment of the relationship between putative C. jejuni C-ring proteins, we analyzed the production and stability of the proteins in whole-cell lysates of wild-type (WT) C. jejuni and mutants lacking the FliF MS ring protein, putative C-ring proteins (FliG, FliM, FliY, and FliN), the FliI ATPase, and the FliH spoke protein. As observed previously, FliF and FliG were dependent on each other for stability but not required for the stability of other proteins (Fig. 2A) (66). FliM, FliH, and FliI were also not required for stability of other MS ring or C-ring proteins. In contrast, FliM levels were reduced and FliN was absent in the C. jejuni Δ*fliY* mutant (Fig. 2A). In the Δ*fliN* mutant, FliY levels were reduced, and FliM levels were only slightly lower than in the WT (Fig. 2A). These findings indicate that FliY and FliN are dependent on each other for full stability, and FliM also requires FliY for stability to a degree. We were unsuccessful in generating antiserum to assess FliI production in C. jejuni.

We next evaluated the contribution of the C-ring proteins to flagellar motility in semisolid Mueller-Hinton (MH) motility agar and in liquid MH broth with or without 0.35% methylcellulose (MC). The addition of MC to MH (MH+MC) broth increases viscosity, which can enhance C. jejuni motility, as the bacterium characteristically swims faster in viscous environments (8, 67, 68). WT C. jejuni was motile in motility agar (Fig. 2B), and approximately 90% of WT C. jejuni cells swam normally, with frequent darting and directional changes in MH and faster swimming with turns in MH+MC broth. The C. jejuni Δ*fliM* and Δ*fliY* mutants were immobile in all media analyzed (Fig. 2B). Although the C. jejuni Δ*fliN* mutant did not swim in motility agar or liquid MH broth, ∼10% of the Δ*fliN* mutant cells showed turning motions in the MH+MC broth, indicating a partially functional motor insufficient to support propulsion. These results support divergent roles of C. jejuni FliY and FliN in flagellar motility, consistent with our phylogenetic analyses (Fig. 1A). The C. jejuni Δ*fliH* and Δ*fliI* mutants were motile in motility agar, although the motile rings were smaller than those in WT C. jejuni (Fig. 2B). Roughly 10% or less of both populations swam normally in MH and MH+MC broth, with the Δ*fliH* mutant displaying a slower velocity of motility than that of WT C. jejuni.

### Structure and composition of the C. jejuni flagellar C ring.

We determined the structure and composition of the C. jejuni C ring by visualizing C rings in deletion mutants by electron cryo-tomography. We acquired more than 150 tomograms of motors from each of WT C. jejuni and isogenic Δ*fliH*, Δ*fliN*, Δ*fliM*, Δ*fliY*, and Δ*fliM* Δ*fliY* mutants, and we subsequently determined their structures by subtomogram averaging.

**FIG 3:**
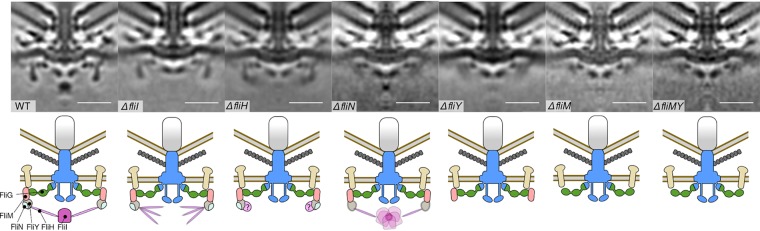
Subtomogram average flagellar motors structures of WT C. jejuni and isogenic mutants. Imaging of WT C. jejuni and Δ*fliI*, Δ*fliH*, Δ*fliN*, Δ*fliY*, Δ*fliM* and Δ*fliM* Δ*fliY* mutant flagellar motors revealed the distal tip of the C ring in its *in situ* functional state. (A) Top row, unfiltered C17 symmetrized subtomogram averages at pixel size of 8.28 Å, 25-nm scale (bar); bottom row, cartoon schematic of C-ring structures, as follows: FliG (green), FliM (pink), FliY (gray), FliN (light blue), FliH and FliI (purple), axial flagellar components (blue), stator complexes (beige), and basal disk (gray disk). Column 1, WT top (7); column 2, Δ*fliI* mutant composed of 205 particles; column 3, Δ*fliH* mutant composed of 187 particles; column 4, Δ*fliN* mutant composed of 281 particles; column 5, Δ*fliY* mutant composed of 155 particles; column 6, Δ*fliM* mutant composed of 187 particles; column 7, Δ*fliM* Δ*fliY* mutant composed of 212 particles. All images are 100-nm by 100-nm boxes.

In the WT C. jejuni flagellar motor, the C ring is intact and well resolved, indicating a complete, full-occupancy structure without substantial flexibility (Fig. 3, column 1). The C ring is at the base of the flagellum, surrounding the FlhA cytoplasmic torus with the ATPase complex below (9, 43). Confirming our previous results, a higher-resolution subtomogram average of the Δ*fliI* mutant motor revealed a loss of FliI density but retained a wild-type C-ring structure (Fig. 3, column 2) (7).

To test the hypothesized role of FliH as a spoke protein that anchors the FliI_6_-FliJ ATPase complex to the fT3SS, we determined a subtomogram average structure of a motor after *fliH* deletion. Consistent with our hypothesis, the Δ*fliH* mutant motor lacked its ATPase complex density (Fig. 3, compare columns 1 to 3), although our resolution was insufficient to resolve FliH itself in our WT motor structure, whose structure is predominantly a narrow coiled-coil beyond the resolution of our images. The Δ*fliH* mutant C ring also maintained its rigidity and resembled the WT C ring, indicating that the C ring does not rely on FliH or FliI for its structural integrity. Curiously, a loss of FliH was accompanied by an additional unidentified density that appeared on the inside face of the C ring that was absent in the Δ*fliI* mutant (Fig. 3, columns 1 to 3). We speculated that removal of FliH disrupted secretion efficiency, leading to an accumulation of abundant chaperoned fT3SS export substrates, such as flagellin bound to its chaperone FliS, on the inner face of the C ring. To test this, we deleted *fliS* in the Δ*fliH* mutant but found that the density remained, refuting our hypothesis (Fig. S2). Next, to test whether FliI was misassembling against the C ring in the absence of FliH, we deleted *fliI* in a Δ*fliH* mutant background, but the density was again unaffected (Fig. S2). Because the unknown density is absent in the Δ*fliI* mutant but present in the Δ*fliI* Δ*fliH* mutant, we propose that the removal of FliH exposes a previously occluded surface that other unknown proteins can bind. These proteins might accumulate in the absence of FliH, although their identity is unclear.

10.1128/mBio.02286-19.2 FIG S2 Examination of the identity of the unidentified C-ring density in the Δ*fliH* mutant. Subtomogram average of flagellar motor structures of C. jejuni Δ*fliH* Δ*fliS* mutant (left column) and Δ*fliH* Δ*fliI* mutant (right column). The top row shows a cross-section through the center of the subtomogram average. The bottom row shows a planar section through the base of the C ring (indicated by white arrow in the side view panel). Arrowheads indicate the position of the unidentified C-ring density that appears in the C. jejuni Δ*fliH* mutant. Download FIG S2, TIF file, 1.9 MB. Copyright © 2019 Henderson et al. 2019 Henderson et al. This content is distributed under the terms of the Creative Commons Attribution 4.0 International license.

The deletion of *fliN* and *fliY* disrupted the structure of the lower rim of the C ring (Fig. 3, columns 4 and 5). The Δ*fliY* mutant flagellar motor showed a disordered lower C-ring rim, as well as loss of the FliI density (Fig. 3, column 5). The Δ*fliN* mutant motor had a similar disorder to the lower C-ring structure but unexpectedly retained a small, yet substantial, FliI density (Fig. 3, column 4). These results suggest two important features of C. jejuni C-ring composition and architecture, as follows: (i) because FliN is unstable in the absence of FliY (Fig. 2A), FliY and probably FliN are components of the C ring, and both are required to stabilize the overall C-ring structure; and (ii) FliY, but not FliN, is a component of the C ring required for anchoring FliI into the C. jejuni motor via FliH. Because of the integration of the FliI ATPase complex into the Δ*fliN* mutant motor but not the Δ*fliY* mutant motor, the C rings of these motors are different in structure and composition, which cannot be discerned by tomography.

The subtomogram average structure of the Δ*fliM* mutant motor showed an additional loss of density compared with Δ*fliN* and Δ*fliY* mutant flagellar motors on the cytoplasm-facing edge of the C ring (Fig. 3, column 6). This observation confirms that FliM forms a ring beneath FliG in the C. jejuni motor. The motor of the Δ*fliM* Δ*fliY* double mutant was comparable to that of the Δ*fliM* mutant motor, indicating that FliM directly contacts FliG to incorporate FliY into the C ring (Fig. 3, column 7). Because the FliI ATPase complex is not integrated into the motors of C. jejuni Δ*fliM*, Δ*fliY*, and Δ*fliM* Δ*fliY* mutants, either FliY alone or together with FliM is likely directly responsible for forming a platform to anchor FliI into the fT3SS via FliH spokes. The remaining densities positioned underneath and running parallel to the membrane may correspond to the globular domains of FliG as in the peritrichous motor (22).

Curiously, we saw a density corresponding to the stator complex component MotB in all mutants (7). In peritrichous motors, stator complexes associate as a function of load into wild-type motors (69–71). This observation further supports our previous speculation that MotB incorporates as a static component into the C. jejuni flagellar motor, irrespective of a functioning motor (7).

### C. jejuni C-ring protein interactions.

Our subtomogram average structures revealed protein locations in the C. jejuni C ring, suggesting protein interactions that may contribute to C-ring assembly. To investigate these possible interactions, we performed *in vivo* coimmunoprecipitation assays with intact C. jejuni cells. To this end, C. jejuni mutants were complemented with N- or C-terminal FLAG-tagged MS ring or C-ring, FliH, or FliI proteins expressed in *trans* from nonnative promoters. Most FLAG-tagged proteins in the respective mutants were expressed at levels similar to those of the native proteins in WT C. jejuni and restored motility, indicating that the FLAG epitope did not hinder function (Fig. S3). The only exception was FliM-FLAG that restored motility slightly to the Δ*fliM* mutant compared to vector alone. Due to our inability to generate an antibody for FliI detection, we could not compare the levels of FLAG-FliI in the Δ*fliI* mutant with native FliI levels in WT C. jejuni.

10.1128/mBio.02286-19.3 FIG S3 *In trans* complementation of C. jejuni mutants with FLAG-tagged proteins. Plasmids expressing N- or C-terminal FLAG-tagged flagellar proteins from promoters to closely replicate expression of the native proteins from WT C. jejuni were introduced into respective C. jejuni mutants. (A) C. jejuni Δ*fliF* mutant expressing FLAG-FliF. (B) C. jejuni Δ*fliG* mutant expressing FliG-FLAG. (C) C. jejuni Δ*fliM* mutant expressing FliM-FLAG. (D) C. jejuni Δ*fliY* mutant expressing FLAG-FliY. (E) C. jejuni Δ*fliN* mutant expressing FLAG-FliN. (F) C. jejuni Δ*fliH* mutant expressing FLAG-FliH. (A to F) Each panel contains an immunoblot of the native protein from a whole-cell lysate of the wild-type strain or mutant strain containing empty vector or the mutant strain expressing the FLAG-tagged protein. An immunoblot is also shown for RpoA in whole-cell lysates to ensure equal loading of proteins across samples. All immunoblots were performed with specific antiserum to each protein. A motility assay is shown in each panel for WT C. jejuni or the mutant strain containing empty vector or the mutant strain expressing the FLAG-tagged protein. Motility was analyzed after 30 h of incubation at 37°C under microaerobic conditions. Download FIG S3, JPG file, 0.1 MB. Copyright © 2019 Henderson et al. 2019 Henderson et al. This content is distributed under the terms of the Creative Commons Attribution 4.0 International license.

**FIG 4:**
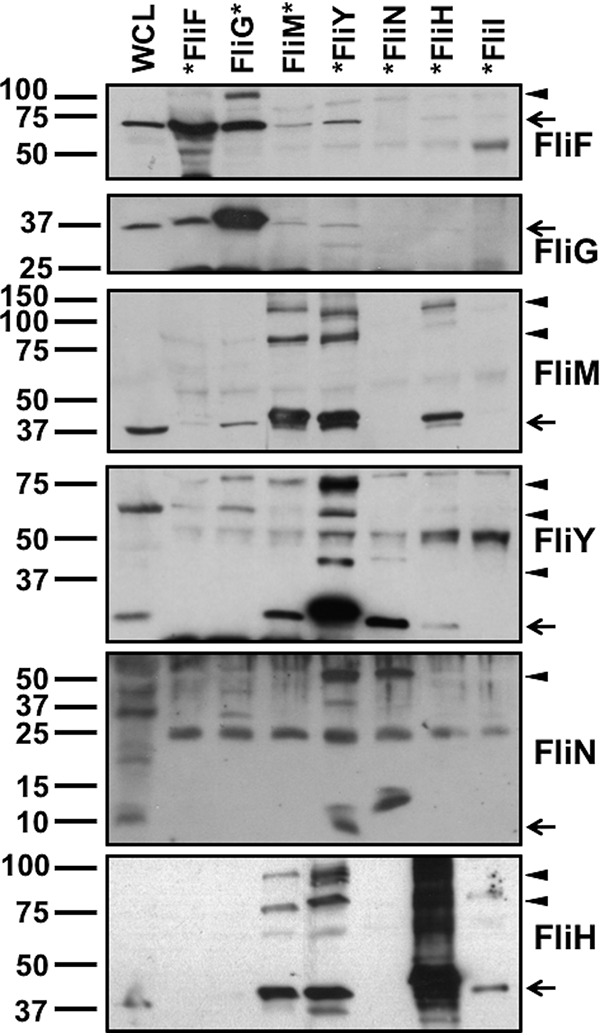
*In vivo* interactions between C. jejuni MS ring and C-ring proteins. N- or C-terminal FLAG-tagged proteins were expressed from plasmids in respective C. jejuni mutants and immunoprecipitated by FLAG tag antibody resin after cross-linking cells by formaldehyde. The position of the FLAG tag is indicated by the position of the asterisk (*) at the beginning or end of each protein that labels each lane at top. Each lane shows the detected proteins that coimmunoprecipitated with the FLAG-tagged protein indicated. Each protein was detected with specific antiserum. A whole-cell lysate (WCL) of WT C. jejuni was run alongside the coimmunoprecipitated proteins to indicate the size and position of the native protein. Arrows indicate the correct size of the monomer for each protein detected in the immunoblot. Arrowheads indicate bands that may represent a complex formed by the FLAG tag-immunoprecipitated protein with other proteins.

Confirming our tomography analysis that FliN is not required for assembly of the FliI ATPase via FliH (Fig. 3), the only protein we detected interacting with FliN was FliY (Fig. 4). We did not detect interactions between FLAG-FliN and FliH or FliM, which are known FliN-interacting partners in peritrichous flagella (19–21, 31, 40, 72–74). We also observed FliN in higher-order complexes (>50 kDa) after coimmunoprecipitation with either FLAG-FliY or FLAG-FliN (Fig. 4). These findings, together with our phylogenetic and cryo-tomography analyses, further support C. jejuni FliN as a C-ring component that interacts only with FliY due to an altered C-ring position and function relative to FliN in model peritrichous motors.

In contrast, proteins that coimmunoprecipitated with FLAG-FliY included FliM, FliN, and FliH (Fig. 4), further implicating FliY occupancy at a central hub position in the C ring enabling FliH binding to integrate the FliI ATPase into the motor and FliN incorporation into the C ring. Furthermore, the FliY-FliM interaction supports our tomography analyses that FliM contributes to stable incorporation of FliY into the side of the C ring and vice versa (Fig. 3). Reciprocal coimmunoprecipitations verified interactions of FliM-FLAG with FliY and FliH and of FLAG-FliH with FliM and FliY (Fig. 4). We also observed higher-order complexes containing FliM, FliY, and FliH after coimmunoprecipitation with any one of these proteins, suggesting that FliM, FliY, and FliH form a heteromeric complex (Fig. 4). Again, both FliM-FLAG and FLAG-FliH did not coimmunoprecipitate FliN. These findings along with the cryo-tomography analysis further suggest that both FliM and FliY contribute to the formation of a platform for FliH binding to anchor the FliI ATPase complex into the motor underneath the fT3SS export gate. Importantly, these findings strongly support C. jejuni FliY replacing the function of FliN in model peritrichous motors. Consistent with other flagellar motors (75–77), FLAG-FliI only interacted with FliH in our assays (Fig. 4), supporting the idea that C. jejuni FliH forms spokes or bridges between a FliM-FliY platform at the lower rim of the C ring and the FliI ATPase complex.

We attempted to detect interactions between the C. jejuni FliG rotor protein and other C-ring proteins. We observed weak interactions between FliG-FLAG and FliM, which is consistent with rotor-switch protein interactions observed in other flagellar systems (Fig. 4). We also observed FliM-FLAG and FLAG-FliY to coimmunoprecipitate a small amount of FliF and FliG (Fig. 4). These results imply that FliM may directly interact with FliG to tether the lower region of the C ring to the rotor, leading to the incorporation of FliY and FliN into the lower region of the C ring.

**FIG 5:**
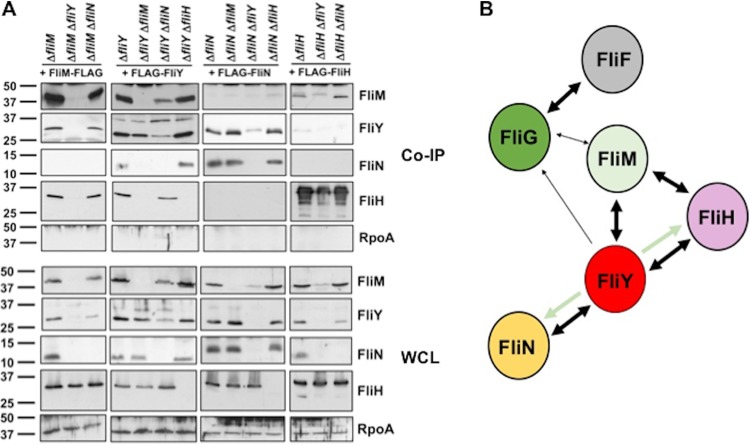
Interdependencies of C-ring proteins on each other for interactions. (A) Coimmunoprecipitation (Co-IP) analysis of C-ring-protein interactions in mutants lacking another C-ring protein. C-terminal FLAG-tagged FliM or N-terminal FLAG-tagged FliY, FliN, or FliH was expressed from a plasmid in C. jejuni single or double mutants lacking specific flagellar proteins and immunoprecipitated by FLAG tag antibody resin after cross-linking cells by formaldehyde. Immunoprecipitated proteins were detected with specific antisera. The top set of immunoblots (Co-IP) shows results from coimmunoprecipitation experiments. The bottom set of immunoblots (WCL) shows levels of proteins in whole-cell lysates of mutants. RpoA is included as a control protein that should not coimmunoprecipitate with a C-ring protein and as a control to ensure equal loading of whole-cell lysates. (B) Summary model of C. jejuni C-ring-protein interactions from coimmunoprecipitation experiments. Double arrows indicate interactions verified by reciprocal coimmunoprecipitation experiments. Single arrows indicate an interaction that could only be observed by immunoprecipitation of one of two interacting partners. Thinner arrows indicate relative weaker interactions. Pale-green arrows indicate that the FliY interactions with FliH and FliN are dependent on FliM.

Some of the interactions inferred from these coimmunoprecipitations may have been direct, while some may require the presence or interaction of a third C-ring protein. To better understand if certain C-ring protein interactions were dependent on a third C-ring protein, we expressed FLAG-tagged proteins in *trans* for coimmunoprecipitation analysis in C-ring double mutants. Due to the dependence of FliM on FliY for stability, we were unable to assess whether FliM-FLAG interacted with FliH independently of FliY (Fig. 5A). Although the level of native FliY was lower in the Δ*fliM* Δ*fliN* mutant than that in the Δ*fliM* mutant, we clearly detected interactions between FliM-FLAG and FliY and FliH, indicating that FliM interacts directly with FliY and FliH independently of FliN (Fig. 5A and B). Thus, these data further demonstrate that FliN in C. jejuni does not contribute to FliI ATPase assembly as in other systems (19–21).

FLAG-FliY required FliM to form a complex with its interacting partner FliN or FliH (Fig. 5A and B). Since FliM does not interact with FliN (Fig. 4), FliM binding to FliY may alter the FliY conformation to enable interaction with FliN. Considering that our tomography analysis indicated that both FliM and FliY are required for FliI ATPase complex incorporation via FliH (Fig. 3), our coimmunoprecipitation results suggest one of the two following possibilities: FliM and FliY together form an interface to interact with FliH and integrate the FliI ATPase into the motor, or FliM binding to FliY alters FliY conformation to enable interactions with FliH, similarly to how FliM binding to FliY likely alters FliY conformation for interactions with FliN (Fig. 5B). Because we observed FLAG-FliY to interact with FliM and FliH in the Δ*fliY* Δ*fliN* mutant (Fig. 5A), FliY appears to be dependent only on FliN for full stability and not for interactions with other C-ring proteins.

Other results from our assays indicated that FLAG-FliN interactions with FliY occurred independently of FliM and FliH, supporting that FliN only binds to FliY (Fig. 5A and B). Also, FliY interacted with FliM and FliN in the absence of FliH (Fig. 5A). These findings support a model where FliM and FliY are major components of the sides of the C ring, possibly together forming an interface to interact with FliH to incorporate the FliI ATPase complex (Fig. 5B). FliN integrates into the C ring by only attaching to FliY, but FliN is important for motility (Fig. 2B and 5B). Finally, our results support the idea that a rigid, well-formed C-ring structure composed of FliM, FliY, and FliN forms in the absence of FliH (Fig. 3).

### Contributions of C. jejuni C-ring proteins to flagellar biogenesis.

**FIG 6:**
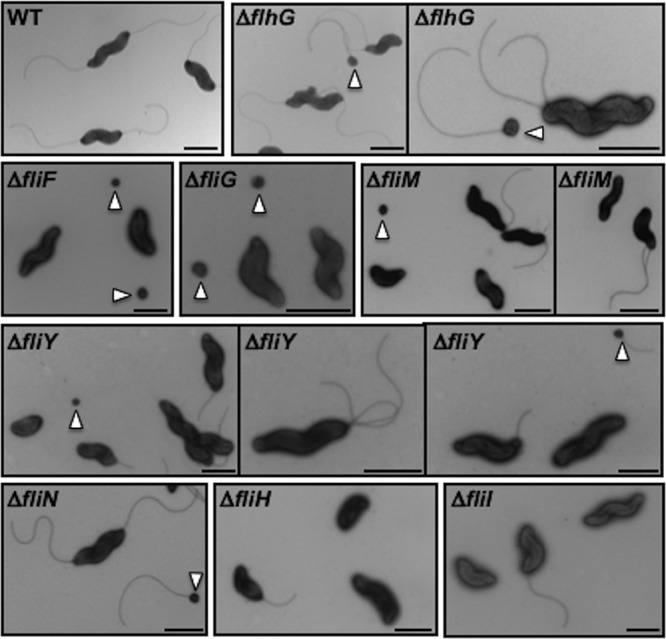
Flagellation phenotypes and minicell production of C. jejuni C-ring mutants. WT C. jejuni and isogenic mutant strains were negatively stained with uranyl acetate and examined by transmission electron microscopy. Arrowheads indicate minicells. Scale bar = 1 μm.

Due to the different composition and architecture of the C. jejuni flagellar C ring relative to those of peritrichous motors, we next investigated the contributions of C. jejuni C-ring proteins to flagellar biogenesis by analyzing flagellation levels and phenotypes of WT C. jejuni and C-ring mutants by transmission electron microscopy (TEM). As a baseline, WT C. jejuni populations contained 89% of the individual cells with what we defined as a “normal” flagellation pattern, amphitrichous flagellation (a single flagellum at both poles; 72% of the population) or a single flagellum at one pole (17% of the population; Fig. 6 and Table 1). The remaining WT cells were aflagellated (8.5%) or hyperflagellated (producing more than one flagellum at least at one pole, at 2%). Due to the lack of σ^54^-dependent flagellar gene expression, all C. jejuni Δ*fliF* and Δ*fliG* mutant cells were aflagellated (Table 1 and Fig. 6) (66).

**TABLE 1:** Quantitative analysis of flagellation phenotypes of individual WT C. jejuni and isogenic mutant cells^*a*^

Flagellation phenotype	% presenting phenotype (mean ± SD) in strain:											
	WT	Δ*flhG*	Δ*fliF*	Δ*fliG*	Δ*fliM*	Δ*fliY*	Δ*fliN*	Δ*fliH*	Δ*fliI*	Δ*fliM* Δ*fliY*	Δ*fliM* Δ*fliN*	Δ*fliY* Δ*fliN*
All cells^*b*^												
Normal^*c*^	89.4 ± 0.8	51.8 ± 5.7	0 ± 0	0 ± 0	32.6 ± 0.7	40.1 ± 0.4	68.6 ± 4.4	12.3 ± 1.1	30.1 ± 4.1	31.0 ± 2.6	34.8 ± 5.3	29.0 ± 4.7
Amphitrichous	72.4 ± 2.5	8.8 ± 0.7			2.9 ± 0.7	4.7 ± 0.2	33.2 ± 3.2	0.3 ± 0.4	1.3 ± 1.9	1.3 ± 0.7	2.6 ± 1.1	0.8 ± 1.1
One at one pole	17.0 ± 3.3	43.0 ± 5.0			29.8 ± 1.3	35.4 ± 0.2	35.4 ± 1.2	11.9 ± 0.6	28.8 ± 6.0	29.7 ± 3.3	32.1 ± 6.4	28.3 ± 3.6
Aflagellated	8.5 ± 1.4	8.4 ± 2.0	100 ± 0	100 ± 0	62.8 ± 0.1	58.0 ± 0.5	21.7 ± 1.9	87.7 ± 1.1	69.5 ± 3.5	65.2 ± 1.6	63.8 ± 4.5	70.2 ± 4.7
Hyperflagellated^*d*^	2.1 ± 0.6	39.8 ± 3.7	0 ± 0	0 ± 0	4.6 ± 0.6	1.9 ± 0.9	9.7 ± 2.5	0 ± 0	0.4 ± 0.6	3.8 ± 0.9	1.5 ± 0.9	0.7 ± 0
Flagellated cells only^*e*^												
Normal^*c*^	97.7 ± 0.6	56.5 ± 5.0			87.7 ± 1.6	95.6 ± 2.1	87.5 ± 3.5	100 ± 0	98.4 ± 2.2	89.7 ± 3.2	95.7 ± 2.8	97.5 ± 0.3
Hyperflagellated^*d*^	2.3 ± 0.6	43.5 ± 5.0			12.3 ± 1.6	4.4 ± 2.1	12.5 ± 3.5	0 ± 0	1.6 ± 2.2	10.9 ± 3.2	4.3 ± 2.8	2.5 ± 0.3

a Two experiments were performed in which >100 individual cells were analyzed for flagellation by transmission electron microscopy.

b Includes data from all cells analyzed regardless of the flagellation phenotype.

c The normal flagellation phenotype includes cells that are amphitrichous (producing a single flagellum at each pole) and cells producing a single flagellum at one pole (“one at one pole”).

d Cells producing 2 or more flagella at least at one pole.

e Only includes the population of flagellated cells, divided into normally flagellated cells (amphitrichous and one flagellum at one pole) and hyperflagellated cells.

Although the C. jejuni Δ*fliM* and Δ*fliY* mutants were nonmotile, 32 to 40% of the cells were flagellated, with a vast majority producing a single flagellum at one pole rather than amphitrichous flagella, indicating that flagellar biogenesis was hindered (Fig. 6 and Table 1). The reduced flagellation phenotypes of these mutants are not due to reduced σ^54^-dependent flagellar rod and hook gene expression, as our previous analysis indicated that C. jejuni Δ*fliM* and Δ*fliY* mutants express these genes at WT levels (66). This level of flagellar biogenesis in the C. jejuni Δ*fliM* and Δ*fliY* mutants is striking, as other motile bacteria are usually aflagellated without FliM (17, 18, 78); the B. subtilis Δ*fliY* mutant is aflagellated, but the Bacillus cereus Δ*fliY* mutant produces flagella (53, 79). In contrast to the C. jejuni Δ*fliM* and Δ*fliY* mutants, more than two-thirds of Δ*fliN* mutant cells were flagellated, with equal proportions producing amphitrichous flagella or a single flagellum (Table 1 and Fig. 6). Again, these findings are striking, as *fliN* mutants in other systems are usually aflagellated (18, 78). The large differences in flagellation levels of the Δ*fliN* mutant compared to those of the Δ*fliY* (and Δ*fliM*) mutant supports our previous tomography and protein interaction analyses that showed that FliN has another function other than being essential for FliI ATPase integration into the motor for protein secretion and flagellation. Because the Δ*fliM* and Δ*fliY* mutants were nonmotile, and only a minority of Δ*fliN* mutant cells showed slight turning motions (Fig. 2B), the flagella produced by these mutants are largely nonfunctional and do not rotate, suggesting that these proteins are important for rotor function.

Of note, C. jejuni double mutants lacking any two of FliM, FliY, or FliN had flagellation levels similar to those of the Δ*fliM* mutant of around 30%, rather than additive effects of the mutations (Table 1 and Fig. 6 and S4A). In each double mutant, FliM, FliY, and FliN proteins were either undetectable or just above the level of detection (Fig. S4B). Thus, the C. jejuni strain producing only the FliG portion of the C ring without significant lower-C-ring structure and the FliI ATPase can produce flagella in a low but significant population of cells. These flagella, however, do not support motility.

10.1128/mBio.02286-19.4 FIG S4 Analysis of C. jejuni C-ring double mutants. (A) WT C. jejuni and isogenic double-mutant strains were negatively stained with uranyl acetate and examined by transmission electron microscopy. Arrowheads indicate minicells. Bar = 1 μm. (B) Immunoblots of MS and C-ring protein levels in whole-cell lysates of WT C. jejuni and isogenic double-mutant strains. A specific antiserum to each C-ring protein or FliH was used to detect each protein. The detection of RpoA served as a control to ensure equal loading of proteins across strains. Download FIG S4, TIF file, 1.1 MB. Copyright © 2019 Henderson et al. 2019 Henderson et al. This content is distributed under the terms of the Creative Commons Attribution 4.0 International license.

We observed that 12% and 30% of the populations of the C. jejuni Δ*fliH* and Δ*fliI* mutants were flagellated, respectively, mostly only with a single polar flagellum produced (Fig. 6 and Table 1), confirming that FliH and FliI are required for optimal flagellar biogenesis. As with the Δ*fliM* and Δ*fliN* mutants, this level of flagellation in the Δ*fliH* and Δ*fliI* mutants is strikingly augmented compared to that in the *fliH* and *fliI* mutants in E. coli and *Salmonella* spp., which are rarely flagellated (47, 48, 78, 80). The flagella produced by the Δ*fliH* and Δ*fliI* mutants are functional, as they supported motility (Fig. 2B). Our findings indicate that the C. jejuni fT3SS functions with fewer parts and has less stringent requirements for its ATPase module than do other species.

### C-ring proteins impact FlhG-dependent numerical control of flagellar biogenesis and spatial regulation of division.

Upon analysis of flagellation in our C. jejuni mutants, we observed noticeable hyperflagellation of certain C-ring mutants. In many polar flagellates, FlhG ATPase orthologs function as numerical regulators of flagellar biogenesis (59, 61, 81–85). As we have previously observed, the C. jejuni Δ*flhG* mutant displayed a hyperflagellation phenotype in 40% of all cells (and 43.5% strictly within the flagellated population; Fig. 6 and Table 1) (59, 61). Within the flagellated Δ*fliM*, Δ*fliN*, and Δ*fliY* mutant populations, the hyperflagellated phenotype was particularly evident. Although Δ*fliM*, Δ*fliN*, and Δ*fliY* mutant flagellation levels were lower than those of the WT (30 to 68% in the mutants versus ∼89% in WT C. jejuni), a greater proportion of mutant flagellated cells were hyperflagellated, with 2- to 5.5-fold higher levels of hyperflagellation relative to those of flagellated WT C. jejuni (Fig. 6 and Table 1). These findings suggest that disruption of parts of the C ring likely impacts FlhG activity for accurate numerical control of flagellar biogenesis.

**FIG 7:**
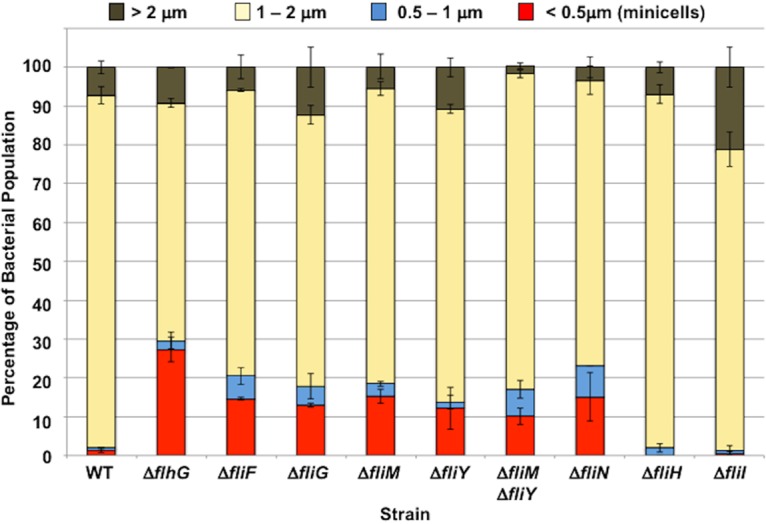
Quantitative assessment of minicell production in C. jejuni MS and C-ring mutants. The lengths of cell bodies of WT C. jejuni and isogenic mutant strains from electron micrographs were measured to determine the percentage of cell populations that are normal size cells or are minicells. Two experiments were performed in which at least 100 individual bacteria were examined per strain. The data are reported as the percentage of bacterial populations with the following cell lengths: >2 μm (brown), 1 to 2 μm (yellow), 0.5 to 1 μm (blue), and <0.5 μm, minicells (red). The data represent the average of the results from the two experiments. Bars represent standard deviations.

FlhG also influences spatial regulation of septal Z ring formation and cell division in C. jejuni (59). In the C. jejuni Δ*flhG* mutant, nonviable minicells form when division occurs at polar regions rather than strictly at the midpoint for symmetrical division (59). We previously discovered that C. jejuni lacking FliF, FliM, and FliN produced an elevated minicell population (59), indicating that flagellar proteins impact FlhG activity related to spatial regulation of division, in addition to flagellar number control. Therefore, we analyzed whether C. jejuni FliY, FliH, and FliI influence FlhG-dependent spatial regulation of division. We measured the lengths of cell bodies of WT C. jejuni and mutant populations by TEM to determine the level of minicells as a proxy for symmetrical division defects. Similar to our prior analysis, about ∼90% of the WT C. jejuni cell bodies were 1 to 2 μm in length, and 7.2% were elongated (<2 μm, Fig. 7) (59). Only 1.3% of the cells were minicells (<0.5 μm in length). In comparison, over 27% of cells were minicells in the C. jejuni Δ*flhG* mutant population (Fig. 6 and 7). Consistent with our previous findings for the C. jejuni Δ*fliF* mutant, the Δ*fliG*, Δ*fliM* and Δ*fliN*, Δ*fliY* mutants produced a similar level of minicells in ∼12% of the population (Fig. 6 and 7) (59). These defects in FlhG-dependent activities for spatial regulation of division are specific for MS ring and C-ring mutants, as minicell production was not evident in the C. jejuni Δ*fliH* and Δ*fliI* mutants (Fig. 7). We only observed an enhanced elongated population in the Δ*fliI* mutant (Fig. 7). These findings further support the idea that C. jejuni C-ring proteins have additional functions outside motility in influencing FlhG-dependent activities for numerical regulation of flagellar biogenesis and spatial regulation of cell division.

### C. jejuni C-ring proteins impact polar localization of FlhG.

**FIG 8:**
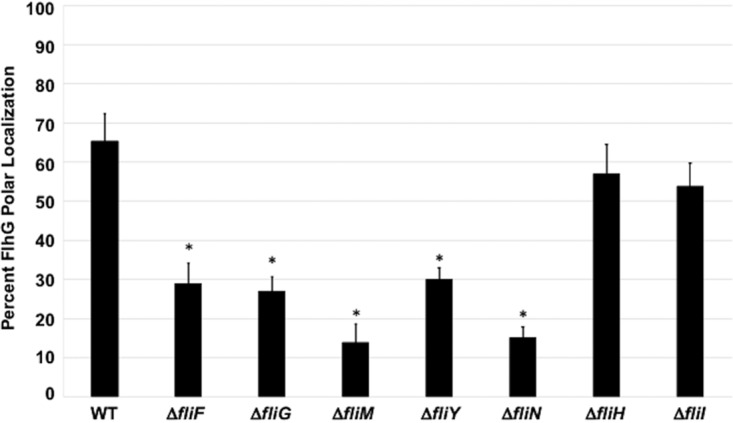
FlhG polar localization in WT C. jejuni and isogenic flagellar mutants. Strains were analyzed by immunofluorescent microscopy after staining with both FlhG and whole C. jejuni antisera. Cells in which detection of FlhG was exclusively at poles were considered positive for polar localization of FlhG. Each strain was analyzed in triplicate, and at least 100 individual cells were counted per sample. After analysis, the percentage of cells with exclusive polar localization of FlhG were averaged, and the standard deviations were determined (bars). A Student's *t* test was performed to determine the statistical significance of differences in polar localization of FlhG between WT and mutant strains (*, *P < *0.05).

Our previous analysis indicated that FlhG with an N-terminal FLAG tag is found predominantly at the poles of C. jejuni cells (61), which is a likely requirement for FlhG to numerically regulate polar flagellar biogenesis and prevent division at the poles. We analyzed whether polar localization of native FlhG was altered in C. jejuni C-ring mutants, which might contribute to understanding how C-ring proteins influence FlhG-dependent activities. Similar to our previous analyses, we found ∼65% of WT C. jejuni cells to position FlhG exclusively at poles (Fig. 8). However, the level of cells with FlhG polarly localized dropped over 50% or more in mutants lacking the FliF MS ring protein or the FliG, FliM, FliY, and FliN C-ring proteins (Fig. 8). In contrast, the level of FlhG polar localization was at WT levels in the Δ*fliH* and Δ*fliI* mutants, which did not display any FlhG-dependent defects in numerical control of flagellar biogenesis or spatial regulation of division (Table 1 and Fig. 7). Our data support the idea that the formation of a structured C-ring structure composed of FliM, FliY, and FliN is required for efficient polar localization of FlhG so that FlhG can properly numerically regulate flagellar biogenesis and spatially regulate placement of the septal Z ring. Combined, our results implicate unique composition and architecture of the flagellar C ring in C. jejuni to impact biological activities beyond flagellar biogenesis and motility.

## DISCUSSION

Flagellar motors across bacterial species have conserved core structures composed of proteins with conserved functions, although recent findings have shown that some motors have recruited extra proteins to form additional structures that adapt and enhance motor function (6–8, 10). Here, we show that the conserved flagellar motor core has also diversified, leading to structural alterations and enabling core components to subdivide existing roles and develop new roles. Using phylogenetic, operonic, genetic, and *in situ* structural analyses, we show that the C. jejuni C ring contains a paralogous duplication of an ancestral FliN-like protein to yield contemporary FliN and FliY, with FliY retaining a function more similar to that of FliN from the well-studied peritrichous E. coli and *Salmonella* sp. motors, and FliN diverging to a more supporting role in preserving C-ring structure for flagellar rotation. The C. jejuni C ring has also evolved to contribute to the localization of FlhG to regulate two important cellular processes in C. jejuni, numerical control of flagellar biogenesis, and spatial regulation of division. Finally, our results also reveal that the C. jejuni fT3SS can function as a secretion machine with fewer parts, being less dependent on its ATPase complex than are other T3SSs.

Our results enable speculation on how the C. jejuni C ring evolved. Our phylogenetic study suggests that an additional copy of an ancestral FliN-like protein evolved in an ancestral epsilonproteobacterium, producing two initially identical SPOA domain-containing proteins that diverged. The fact that both FliY and FliM feature CheC domains suggests that the ancestral sequence had both CheC and SPOA domains and that the CheC domain was subsequently lost by FliN proteins. Although we cannot rule out a horizontal gene transfer origin, a gene duplication is more plausible, because an exogenous homolog with a diverged binding interface is not guaranteed to be able to assemble into a preexisting structure, whereas an identical duplicate will by definition assemble. Furthermore, the spirochete FliY/FliN families show a similarly deep branching pattern. The divergence of the two ancestral FliN-like proteins gave rise to contemporary C. jejuni FliY and FliN. We propose that contemporary FliY retained the classical FliN role exemplified by FliN from peritrichous flagellar motors based on our finding that FliY is located in an operon with FliM, C. jejuni FliY has higher sequence similarity to FliN of peritrichous flagellar motors, FliY and FliM are required for the assembly of FliH to anchor the ATPase complex, and FliN is less critical for flagellar biogenesis than is FliY. We propose that FliN, meanwhile, neofunctionalized as a structural stabilizer. Sequence drift led to a loss of binding between FliN and FliM, necessitating that FliY serve as a bridge between them; this shift of FliY and FliN from homooligomer to obligate heterooligomer is seen in other heterooligomeric structures that have evolved from paralogous duplications (86, 87). Sequence drift also led FliN to lose an ability to bind FliH, as this function was fulfilled by FliY.

Whether the ancestral FliN-like protein acquired its CheC domain or whether the ancestor already had a CheC domain is unclear. It is possible that FliM represents the ancestral state that included a CheC domain and that the CheC-less FliN proteins have selectively lost their CheC domains. Indeed, the loss of a CheC domain requires evolution only of an alternative translation start site, whereas the gain of a CheC domain requires correct relative positioning of the donor CheC protein in the genome, arguing against multiple independent CheC acquisitions. Although we do not know the origins of this second allele that encodes contemporary FliN, *cjj81176_0374*, the gene upstream of *fliN* in the C. jejuni strain 81-176 genome, encodes a protein of unknown function with a predicted distantly related CheC domain echoing the typical FliY and FliM arrangement of an N-terminal CheC-like domain, followed by a C-terminal SPOA domain. This may be a relic of an ancestral state that included the CheC domain. Alternatively, it may represent an ancestral state prior to fusion of the two proteins, where the original *fliY* allele may have duplicated and integrated elsewhere in the genome before point mutations fused the CheC-like domain with the SPOA domain.

**FIG 9:**
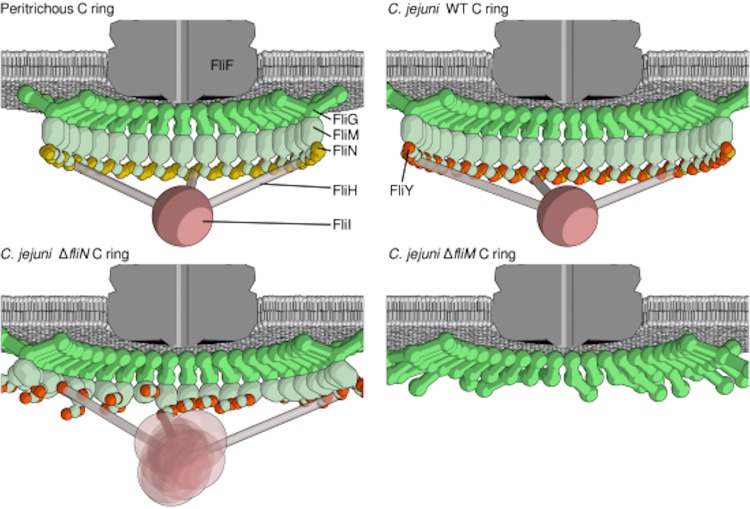
Model of C-ring assembly, composition, and function in C. jejuni. Cartoon schematics show the architecture of the peritrichous C ring (top left) and the C. jejuni C ring (top right), the effect of deletion of *fliN* partially disrupting the C-ring and ATPase complex architecture (bottom left), and the effect of deletion of *fliM* further disrupting the C-ring architecture (bottom right).

Our data enable us to propose a model for the architecture of the C. jejuni C ring whereby FliM and FliY form a structural and functional module of the C ring through their SPOA domains. FliN and FliY are often mutually exclusive, and B. subtilis FliY can restore motility to a *Salmonella* sp. *fliN* mutant (53), indicating that FliY and FliN are equivalent components of the lower rim of the C ring in different species together with FliM. Our findings show that FliN and FliY are not exchangeable in the C. jejuni C ring and possess distinct roles, whereby FliY likely forms a platform with FliM for FliH binding and anchoring the ATPase complex into the fT3SS, and FliN stabilizes a concrete C-ring architecture between the SPOA domains of FliY and FliM. Our findings suggest that FliN and FliY oligomerize with FliM differently in different species. Incorporating our results into the archetypical model from E. coli and *Salmonella* sp. motor of the lower rim of the C ring as a circularized spiral structure of SPOA domains formed by multimerization of a FliM-FliN_3_ ternary complex enables us to propose a model for the architecture of the elaborated C. jejuni C ring (Fig. 9). Our interaction data led us to propose that the SPOA domains of FliM, FliY, and FliN retain a common circularized helical architecture but with complexes forming a FliM-FliY-FliN-FliY repeat through SPOA domain interactions (Fig. 9). This arrangement would allow FliM and FliY to form an interface for FliH binding and FliI integration (even in the absence of FliN, albeit with a more disordered architecture), with FliN providing interlocking stabilizers between FliY proteins to bring adjacent complexes together (Fig. 9). While is unclear where the CheC domain from FliY fits into this model, and this model fails to incorporate previous observations of symmetric SPOA dimers for FliY, this model is the most parsimonious with our data. Our model provides a framework for future exploration to unravel how this unusual collection of proteins in a bacterium form a functional C ring that is different from that in other bacteria.

Observations from the closely related epsilonproteobacterium H. pylori C-ring components have demonstrated some similar interactions that support our proposed C-ring architecture (57). Structural analysis of H. pylori FliM, FliY, FliN, and FliH suggests that an alteration of C-ring architecture and function has also occurred in this species (56, 57). Nevertheless, H. pylori FliY-FliM and FliY-FliN heterodimers can interact with FliH *in vitro*, which is different from what we observed *in vivo* in C. jejuni with only FliM and FliY contributing to FliH interactions. H. pylori also appears to have different requirements of C-ring proteins for flagellar assembly from those to C. jejuni. H. pylori *fliM* and *fliY* mutants are severely defective for flagellar biogenesis, but a *fliN* mutant is much less so; all of these H. pylori mutants are generally less flagellated than are the respective C. jejuni mutants (56, 57, 88). Our work combined with this analysis in H. pylori, a closely related epsilonproteobacterium, demonstrate that flagellar motors are composed of different components across bacterial species, which result in motors with different assemblies of substructures, alteration of motor output for propulsion, and roles outside flagellar motility.

Our model suggests an explanation for why, despite flagellation of the C. jejuni Δ*fliM*, Δ*fliY*, Δ*fliN* mutants, none of these strains are motile. These nonmotile mutants predominantly assembled a single flagellum at one pole, a flagellation pattern similar to those of the motile C. jejuni Δ*fliH* and Δ*fliI* mutants, indicating that the lack of motility in the Δ*fliM*, Δ*fliY*, and Δ*fliN* mutants is not due to single flagellation. The lower portion of the C ring appears to be less structured in these mutants in our tomography analysis, indicating that all three proteins are required to form a rigid C ring. We propose that disruption of this region of the C ring has consequences for FliG folding and/or assembly into the rotor for full activity. That the disruption of C ring structure has significant consequences for motility and assembly highlights an interesting apparent paradox in C-ring biology. Studies in E. coli have shown that FliM and FliN are not static components of the C ring but rather bind and dissociate dynamically. These dynamics are not detrimental to motor function in this species (89–92). However, the deletion of FliM, FliY, and FliN is highly damaging to the C. jejuni C ring. It is possible that C. jejuni C-ring components, like its stator complexes, are static components of the C ring, especially since we discovered that these proteins are important for FlhG-dependent cellular processes that can affect motility and division impacting *in vivo* fitness.

One remaining question is how the C. jejuni flagellar C ring impacts FlhG localization for activity to accurately control flagellar number and prevent division at the poles. All MS and C-ring mutants (but not *fliH* or *fliI* mutants) are defective in spatial regulation of division, and mutants lacking FliM, FliY, and FliN are hyperflagellated, demonstrating defects in both known functions of FlhG and implicating the C ring as contributing to FlhG biological activity. The levels of hyperflagellation and division defects are not as high, however, as those of the C. jejuni Δ*flhG* mutant, suggesting that other factors also influence FlhG activity. Currently, it is unknown how FlhG localizes to poles in C. jejuni. FliF and FliG like do not directly influence FlhG localization, as C. jejuni mutants lacking these proteins do not incorporate FliM, FliY, or FliN into the C ring. Thus, either complete formation of the C ring by FliM, FliY, or FliN or integration of one of these specific proteins impacts FlhG localization. As we have yet to detect a stable interaction of FlhG with any C-ring protein, the formation of a structured flagellar C ring (regardless of presence of FliH or FliI) may indirectly influence FlhG polar localization. In Vibrio cholerae, the HubP protein is involved in the polar localization of FlhG and other MinD/ParA ATPase homologs involved in chromosome segregation and localization of the chemotactic machinery (93). However, C. jejuni lacks HubP. We previously showed that FlhG has a C-terminal amphipathic helix (61); in related MinD proteins, this amphipathic helix recognizes polar curvature and inserts into the cytoplasm-inner membrane interface upon MinD binding ATP (94–97). One possibility is that C. jejuni C-ring formation influences the curvature or distortion of membrane phospholipids that is required for FlhG polar localization. Alternatively, the C ring could interact with a protein that directly interacts with FlhG for polar localization. Regardless of the mechanism, formation of the flagellar C ring likely serves as a polar determinant for polar localization of FlhG so that it can regulate flagellar number and spatial control of septal Z ring formation for accurate symmetrical division.

Our results highlight remarkable differences in the C. jejuni fT3SS to function adequately even without its ATPase complex, which is usually critical for T3SS function. Although mechanisms of T3SS are not completely clear, it is thought that ATP hydrolysis by the FliI ATPase largely functions to dissociate substrates from chaperones for delivery to the fT3SS export gate, whereas the proton motive force powers substrate secretion through the export gate (47, 48). *Salmonella fliM* or *fliN* mutants are aflagellated, presumably due to failure to assemble the FliI ATPase underneath the fT3SS export gate via C-ring-FliH interactions (17, 18, 40, 78). Furthermore, *Salmonella fliH* and *fliI* mutants rarely synthesize flagella (47, 48, 80). Disrupting FliI assembly into the C. jejuni motor, however, still allowed for a substantial proportion of flagellated cells (∼12 to 40% in the *fliH*, *fliM*, *fliI*, and *fliY* mutants). To our knowledge, the relative levels of organelle biogenesis in these C. jejuni mutants are much higher than usually reported in analogous mutants in other motile bacteria and in related injectisome T3SS mutants. These findings point to a striking minimalism for a functional fT3SS in C. jejuni. It is unclear what may be different about C. jejuni or its fT3SS to enable flagellar assembly with a minimal fT3SS machinery. *Salmonella* C-ring mutants can partially overcome secretion defects with elevated production of FliI or secretion substrates, with increased FliI thought to augment chaperone-free substrates or increase the activation and opening of the fT3SS export gate (1, 47, 48, 50, 98). It is possible that the C. jejuni fT3SS export gate has a naturally more open conformation, is more easily accessible by secretion substrates, or is operated by a protein motive force that is naturally higher in C. jejuni and can compensate fairly well in the absence of FliI. Alternatively, C. jejuni substrates may be produced at higher levels than in other bacteria or may be less tightly associated with chaperones for easier delivery into the fT3SS export gate. C. jejuni does use its fT3SS to also secrete Fed and Cia proteins that are not required for motility but for commensal colonization of chickens and interactions with human intestinal cells (99–104). C. jejuni may have developed an fT3SS with enhanced mechanics to secrete a specific but broader range of substrates than for other fT3SSs to be more competitive for *in vivo* fitness. A full understanding for how the C. jejuni fT3SS can moderately function without an ATPase complex will require a deeper understanding of how flagellar substrates are concentrated and delivered to the fT3SS export gate, whether flagellar secretion is powered differently, and the exact contribution of the FliI_6_-FliJ ATPase complex to secretion in C. jejuni.

An intriguing question to consider regarding C-ring composition and function is what were the evolutionary steps in developing the C-ring structure of *Campylobacter* species and how the C ring acquired functions to influence flagellar biogenesis, rotor function, and FlhG-dependent activities related to flagellar number control and spatial regulation of division. Because flagellar motors are widespread and likely occurred early in evolution, primordial functions of the C. jejuni C-ring proteins were for flagellar biogenesis and motility. We expect that the impact of flagellar C-ring proteins on FlhG-dependent activities likely occurred later and are specific for *Campylobacter* species and maybe a few other amphitrichous bacteria. We suspect that the complex composition of the C. jejuni flagellar C ring is now favorably maintained due its expanded role in the biology of C. jejuni. Our results highlight, again, that there is no one canonical flagellum; rather, the bacterial flagellum is a set of diverse evolved types and subtypes with many nuanced differences.

## MATERIALS AND METHODS

### Bacterial growth and media.

The C. jejuni 81-176 strain is typically grown under microaerobic conditions (85% N_2_, 10% CO_2_, 5% O_2_) on Mueller-Hinton (MH) agar at 37°C. For routine growth to perform most experiments, C. jejuni strains were grown from frozen stocks for 48 h under microaerobic conditions at 37°C and then restreaked onto MH agar and grown for an additional 16 h under identical conditions. As required, antibiotics were added to the MH medium at the following concentrations: 10 μg/ml trimethoprim (TMP), 15 μg/ml chloramphenicol, 50 μg/ml kanamycin, and 0.5, 1, 2, or 5 mg/ml streptomycin. All C. jejuni strains were stored at –80°C in a solution of 85% MH broth and 15% glycerol. E. coli DH5α, XL1-Blue, and BL21 strains were grown on Luria-Bertani (LB) agar or in LB broth containing the following concentrations of antibiotics when appropriate: 100 μg/ml ampicillin, 50 μg/ml kanamycin, or 15 μg/ml chloramphenicol. All E. coli strains were stored at –80°C in a solution of 80% LB broth and 20% glycerol.

### Construction of mutants.

All methodologies to construct plasmids and C. jejuni mutants are described in Text S1 in the supplemental material. All bacterial strains and plasmids used in this study are listed in Tables S1 and S2.

10.1128/mBio.02286-19.6 TABLE S1 Bacterial strains used in this study. Download Table S1, PDF file, 0.1 MB. Copyright © 2019 Henderson et al. 2019 Henderson et al. This content is distributed under the terms of the Creative Commons Attribution 4.0 International license.

10.1128/mBio.02286-19.7 TABLE S2 Plasmids used in this study. Download Table S2, PDF file, 0.1 MB. Copyright © 2019 Henderson et al. 2019 Henderson et al. This content is distributed under the terms of the Creative Commons Attribution 4.0 International license.

### Phylogenetics of FliM, FliN, and FliY.

Thirty-eight protein sequences of FliM, FliN, and FliY from 12 organisms were used for this analysis. Initial multiple-sequence alignments were conducted using the package BAli-Phy. The aligned sequences were evaluated using a transitive consistency score (TCS) in the suite T-Coffee to remove residues with a value lower than 3. SeaView was used to remove gap sites in the alignment before scripts were used to trim the sequence alignment to the final 74 residues, which corresponds to the approximate common SPOA domain present in FliM, FliN, and FliY. Phylogenetic trees were generated using a maximum likelihood method in the package Garli. One thousand bootstrap repeats were generated with values for genthreshfortopoterm of 100,000 and significanttopochange of 0.00001. Operons for each organism were generated through custom scripts utilizing the python package Biography. Initial diagrams were produced showing the relative genomic location of each gene; these were manually edited and annotated with the presence of full and degenerate EIDAL motifs.

### Electron microscopy sample preparation, tilt-series data collection, and tomographic data processing.

C. jejuni was grown microaerobically for 48 h on MH agar at 37°C prior to restreaking on fresh plates for 16 h. Overnight cell cultures were resuspended from plates in ∼1.5 ml of MH broth, spun down to an optical density at 600 nm (OD_600_) of 25, and combined with gold fiducial markers. Cell suspensions (3 μl) were applied to glow discharged Quantifoil R2/2 grids (200 mesh), blotted, and plunge frozen in a liquid ethane-propane mixture using the Vitrobot Mark 4 robot (FEI Company). The blotting conditions used were as follows: blot force, 2; blot time, 2; drain time, 1; humidity, 95%; H-value, 0.65; and no wait time. Grids were stored under liquid nitrogen until data collection.

Data were collected on a 200-kV Twin F20 microscope (FEI Company) using a Falcon II direct electron detector camera (FEI Company) with a Gatan 626 cryo-holder. The software package Leginon was used to record tilts between +54 and −54, beginning at +24 and collecting through to −54 before collecting the remainder of the tilts. The collection magnifications used were 25,000× (Δ*fliY* and Δ*fliN* mutants) for a final pixel size of 8.280 Å and 29,000× (Δ*fliM* and Δ*fliM* Δ*fliY* mutants) for a final pixel size of 7.002 Å. All structures were resized to a pixel size of 8.281 Å for comparison with the WT. A different defocus value was applied to each sample during collection, as follows: Δ*fliY* mutant, 3.5 μm; Δ*fliM* mutant, 2 μm; Δ*fliM* Δ*fliY* mutant, 2.5 μm; and Δ*fliN* mutant, 6 μm. Overnight data collection was enabled by the use of a 3-liter dewer for the cryo-trap and a nitrogen-dispensing pump controlled via Leginon to maintain cryo-holder temperatures.

Tomograms were reconstructed automatically using the RAPTOR software and the package IMOD. All data were low-pass filtered to 3.5 nm. Motors within tomograms were delineated by two points along their rotational axis. The PEET package within IMOD was then used for particle extraction, alignment, and averaging. A reference-free alignment approach with a spherical mask was used for the initial averaging, and this initial average was then used as a reference alongside a mask which excludes the inner and outer membranes from the alignment to generate the final subtomogram averages.

### Motility assays.

C. jejuni strains were suspended from plates in MH broth and diluted to an OD_600_ of 0.8. Each bacterial strain was stabbed into semisolid MH motility medium containing 0.4% agar using an inoculation needle and then incubated for 30 h at 37°C under microaerobic conditions. When appropriate, motility agar contained chloramphenicol or kanamycin to maintain plasmids for in *trans* complementation of mutants. For dark-field microscopy, the cultures were further diluted 1:10 in MH broth or MH broth with 0.35% methylcellulose. Strains were immediately analyzed for motility by applying 3 μl of culture between a glass slide and glass coverslip.

### *In vivo* immunoprecipitation of C. jejuni proteins.

Immunoprecipitation of proteins from C. jejuni for protein interaction studies were performed as previously described, with slight modifications (66, 105). C. jejuni mutant strains expressing appropriate FLAG-tagged proteins from plasmids were grown on six MH agar plates with appropriate antibiotics for 16 h at 37°C under microaerobic conditions. Bacteria were suspended from each plate in 2 ml of phosphate-buffered saline (PBS), centrifuged for 6,000 rpm for 10 min, and resuspended in 6 ml of PBS. Bacteria were cross-linked by the addition of 16.2 μl of 37% formaldehyde (final concentration, 0.1%) and incubated for 30 min at 37°C. Cross-linking was quenched with the addition 1.2 ml of 1 M glycine, followed by rocking for 10 min at 25°C. Bacteria were centrifuged at 6,000 rpm for 10 min. For osmotic lysis of bacterial cells, pellets were resuspended in 1.5 ml 200 mM Tris (pH 8.0), and then the following solutions were added in order with vortexing: 3 ml of 200 mM Tris (pH 8.0) with 1 M sucrose, 0.3 ml of 10 mM EDTA, 0.3 ml lysozyme (10 mg/ml), 9 ml H_2_O, and 0.9 ml 100 mM phenylmethylsulfonyl fluoride (PMSF) (105). After incubation on ice for 15 min, 15 ml of solubilization solution (50 mM Tris [pH 8.0], 10 mM MgCl_2_, 2% Triton X-100) was added. Samples were incubated on ice for 45 min and then centrifuged at 10,000 rpm for 20 min. The supernatant was recovered, and 45 μl of anti-FLAG M2 affinity gel resin (Sigma-Aldrich) was added to immunoprecipitate FLAG-tagged proteins along with any interacting proteins. The lysate was then rocked overnight at 4°C on a nutator. The resin was pelleted by centrifugation at 4°C at 6,000 rpm for 20 min. The resin pellet was washed three times with 3 ml of RIPA buffer (50 mM Tris [pH 8.0], 150 mM NaCl, 0.1% SDS, 0.5% sodium deoxycholate, 1% Triton X-100), with a centrifugation step of 6,000 rpm for 15 min between each wash. The resin was then resuspended in 255 μl SDS-PAGE loading buffer and boiled for 5 min, and 30 μl was analyzed by SDS-PAGE, followed by immunoblotting with specific antisera.

### Immunoblotting analysis.

The procedures for the construction of plasmids for recombinant protein expression, purification of recombinant proteins, and antiserum generation are described in the supplemental material. Preparation of C. jejuni strains for whole-cell lysates (WCLs) was performed as previously described (106). All immunoblotting analysis of WCLs or immunoprecipitated proteins was performed with equal amounts of samples from C. jejuni strains after SDS-PAGE by standard procedures. For the specific detection of proteins in WCLs, 10 μl of WCLs was analyzed to detect FliF and FliG, 12.5 μl of WCLs was analyzed to detect FliM, FliY, and RpoA, 25 μl of WCLs was analyzed to detect FliN, and 4 μl of WCLs was analyzed to detect FliH. Proteins were detected with specific murine, rabbit, or guinea pig antiserum generated previously or as described above. Primary antisera were used at the following concentrations: FliF M204, 1:2,500; (66); FliG M162, 1:2,500; (66); RpoA M251, 1:3,000; (107); FliM M186, 1:2,500; FliY M197, 1:4,000; (66), FliH GP222, 1:6,000; and FliN GP204, 1:500. All primary antisera were applied to the immunoblots for 1 to 2 h, except for the FliN antiserum, which was applied overnight. Appropriate horseradish peroxidase-conjugated goat antibodies were used as secondary antibodies to develop immunoblots.

### Transmission electron microscopy.

C. jejuni strains were resuspended from MH agar into PBS, pelleted for 3 min at 13,200 rpm in a microcentrifuge, resuspended in 2% glutaraldehyde in 0.1 M cacodylate, and then incubated on ice for 1 h. Copper-coated Formvar grids were negatively glow discharged, and bacterial samples were then applied to the grids. The samples were stained with 2% uranyl acetate and visualized with an FEI Tecnai G2 Spirit BioTWIN transmission electron microscope. Flagellar numbers were determined from at least 100 individual cells and averaged from two biological replicates to determine the proportion of bacterial populations producing different flagellation phenotypes, as follows: hyperflagellated (a bacterium producing two or more flagella at one or both poles), wild type (producing a single flagellum at each pole or a flagellum at one pole with the other pole aflagellated), or aflagellated (lacking a flagellum). For analysis of cell body lengths, bacterial cell bodies from electron micrographs were visualized, measured using the ImageJ software, and placed into one of the following four categories: <0.5 μm (minicells), 0.5 to 1 μm, 1 to 2 μm, and >2 μm. After averaging, the standard deviation for each population was calculated.

### Immunofluorescent microscopy.

WT C. jejuni and isogenic mutants were prepared for immunofluorescent microscopy to determine the localization of FlhG, as previously described (61). After fixation and blocking of samples on chamber slides, 70 μl of a mixture containing a 1:50 dilution of FlhG GP188 antiserum or whole C. jejuni UT527 antiserum in 3% bovine serum albumin (BSA), 1% Zwittergent, 0.1% Triton X-100, and 0.02% sodium azide in PBS was applied to each sample and incubated for 30 min at room temperature. Samples were then washed three times with blocking buffer. Seventy microliters of a mixture containing a 1:200 dilution of the appropriate secondary antibody (goat Texas Red-conjugated anti-rabbit antibody or goat isothiocyanate-conjugated anti-guinea pig antibody; Santa Cruz Biotechnology) was added to the sample for 30 min at room temperature. Samples were washed three times with blocking buffer, chamber gaskets were then removed, and two drops of Vectashield (Vector Laboratories) were added, followed by a coverslip. Slides were cured at room temperature overnight before imaging on an Applied Precision PersonalDV deconvolution microscope with an Olympus 100× lens objective and a CoolSNAP-HQ2 camera. Images were adjusted for brightness and contrast using the ImageJ software. Three separate samples were prepared for each strain, and at least 100 cells were counted for each sample. Individual cells were considered positive for FlhG polar localization if the fluorescent signal was observed only at one or both poles. After averaging, the standard deviation for each population was calculated. Student's *t* test was used to evaluate the statistical significance of differences in the levels of polar localization of FlhG between the WT and mutant strains.

All data and methodologies are available upon request.

### Data availability.

Subtomogram averages are available in EMDB (Δ*fliN*, EMD-10341; Δ*fliY*, EMD-10342; Δ*fliM*, EMD-10343; Δ*fliM* Δ*fliY*, EMD-10345; Δ*fliH*, EMD-10454; Δ*fliH* Δ*fliI*, EMD-10455; Δ*fliH* Δ*fliS*, EMD-10456; Δ*fliI*, EMD-10457).

10.1128/mBio.02286-19.5 TEXT S1 Additional Materials and Methods. Download Text S1, PDF file, 0.1 MB. Copyright © 2019 Henderson et al. 2019 Henderson et al. This content is distributed under the terms of the Creative Commons Attribution 4.0 International license.
