# Supplementary figures and images for: Diversification of Campylobacter jejuni Flagellar C-Ring Composition Impacts Its Structure and Function in Motility, Flagellar Assembly, and Cellular Processes
Source: mBio. 2020 Jan 7;11(1):e02286-19. doi: 10.1128/mBio.02286-19 (PMC6946799; doi:10.1128/mBio.02286-19)

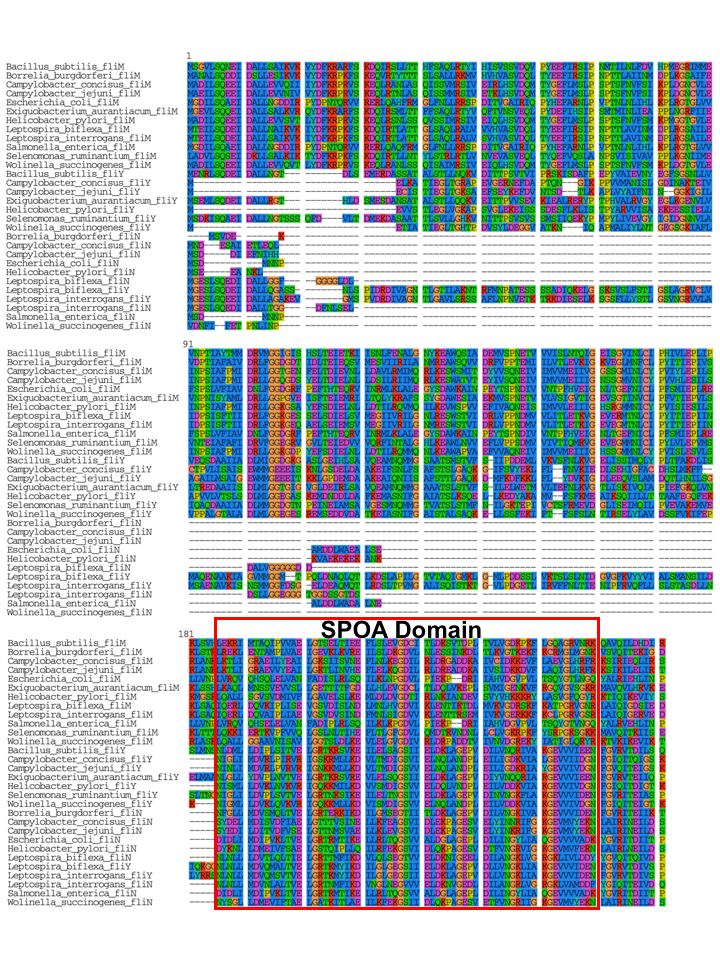

Supplement: FIG S1 [file mBio.02286-19-sf001.tif]

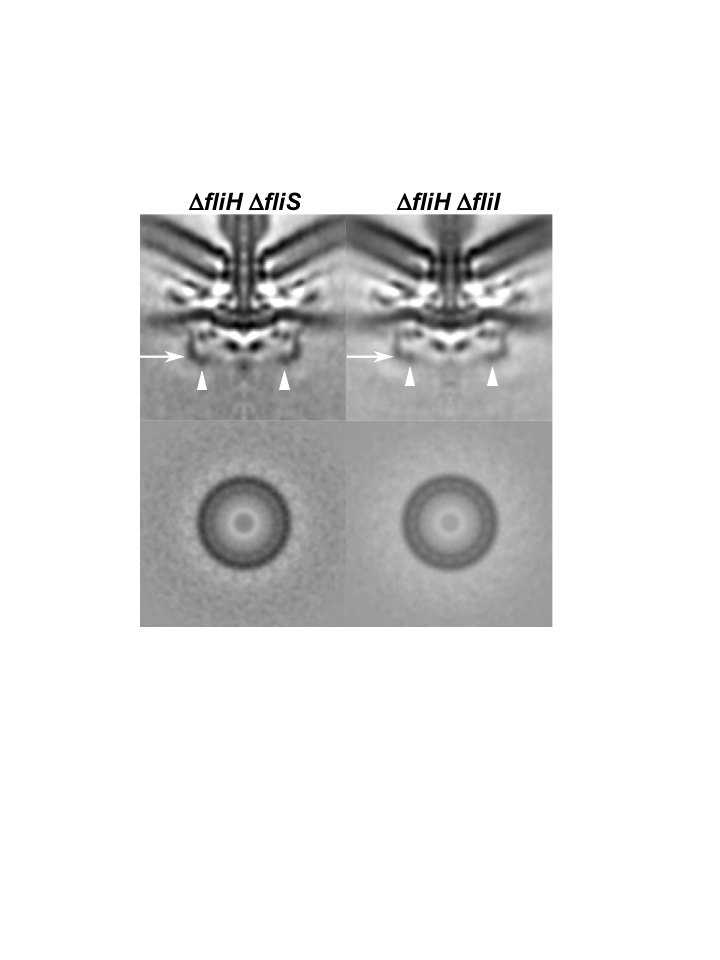

Supplement: FIG S2 [file mBio.02286-19-sf002.tif]

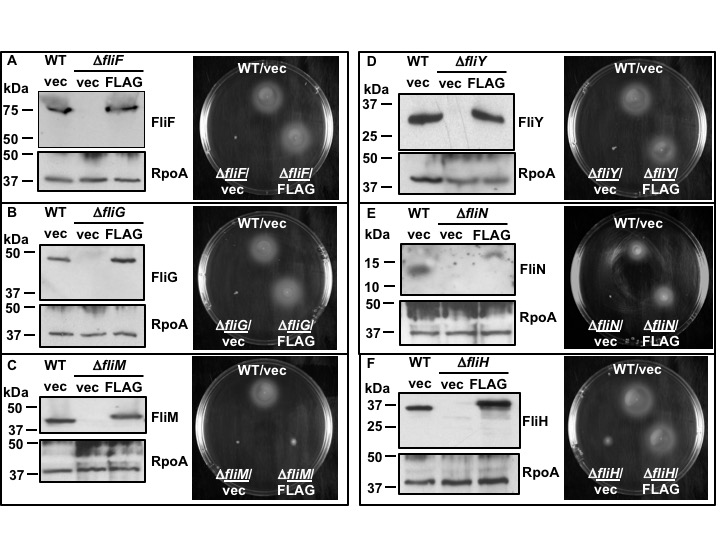

Supplement: FIG S3 [file mBio.02286-19-sf003.jpg]

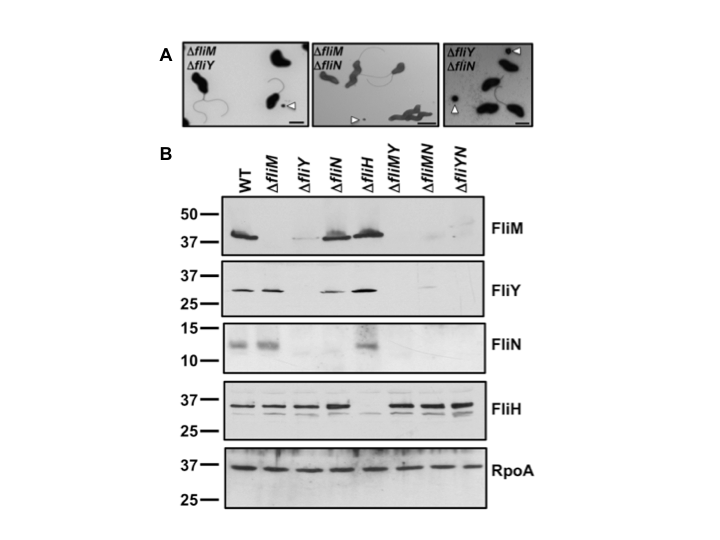

Supplement: FIG S4 [file mBio.02286-19-sf004.tif]
