# Supplementary material for: Diversification of Campylobacter jejuni Flagellar C-Ring Composition Impacts Its Structure and Function in Motility, Flagellar Assembly, and Cellular Processes
Source: mBio. 2020 Jan 7;11(1):e02286-19. doi: 10.1128/mBio.02286-19 (PMC6946799; doi:10.1128/mBio.02286-19)
Supplement: TABLE S1 [file mBio.02286-19-st001.pdf]

Table S1. Bacterial strains used in this study

Strain	Genotype	Source/Reference
DH5α	*E. coli supE44* Δ*lacU169* (φ80l*acZ*DM15) *hsdR17 recA1 endA1 gyrA96 thi-1 relA1*	Invitrogen
DH5α/pRK212.1	DH5α with conjugation transfer element	(3)
BL21 (DE3)	*E. coli fhuA2 [lon] ompT gal (λ DE3) [dcm]* Δ*hsdS λ DE3 = λ sBamHIo* Δ*EcoRI-B int::(lacI::PlacUV5::T7 gene1) i21* Δ*nin5*	New England Biolabs
XL1-Blue	F'::Tn*10 proA*$^{+}$*B*$^{+}$*lacI*^{q} Δ*(lacZ)M15/recA1 endA1 gyrA96* (Nal^{r}) *thi hsdR17* (r_K^- m_K^+) *glnV44 relA1 lac*	(4)
DRH212	81-176 *rpsL*^{Sm}	(1)
DRH2077	81-176 *rpsL*^{Sm} Δ*fliF*	(5)
DRH2257	81-176 *rpsL*^{Sm} Δ*astA* Δ*fliI*	(6)
DRH3302	81-176 *rpsL*^{Sm} *fliM*::*cat-rpsL*	This study
DRH5332	81-176 *rpsL*^{Sm} Δ*fliH fliI*::*cat-rpsL*	This study
DRH6363	81-176 *rpsL*^{Sm} Δ*fliM*/pDRH6346	This study
DRH6411	81-176 *rpsL*^{Sm} Δ*fliH fliS*::*cat-rpsL*	This study
DRH6432	81-176 *rpsL*^{Sm} Δ*fliM* Δ*fliN*	This study
DRH6478	81-176 *rpsL*^{Sm} Δ*fliM* Δ*fliY*/pDRH6346	This study
DRH6509	81-176 *rpsL*^{Sm} Δ*fliY*/pDRH6472	This study
DRH6521	81-176 *rpsL*^{Sm} Δ*fliH*/pDRH6456	This study
DRH6529	81-176 *rpsL*^{Sm} Δ*astA* Δ*fliI*/pDRH6458	This study
DRH6542	81-176 *rpsL*^{Sm} Δ*fliM* Δ*fliY*/pDRH6472	This study
DRH6609	81-176 *rpsL*^{Sm} Δ*fliY fliN*::*cat-rpsL*	This study
DRH6630	81-176 *rpsL*^{Sm} Δ*fliY* Δ*fliN*	This study
DRH6664	81-176 *rpsL*^{Sm} Δ*fliM* Δ*fliN*/pDRH6461	This study
DRH6661	81-176 *rpsL*^{Sm} Δ*fliM* Δ*fliN*/pDRH6346	This study
DRH6670	81-176 *rpsL*^{Sm} Δ*fliY* Δ*fliN*/pDRH6472	This study
DRH6673	81-176 *rpsL*^{Sm} Δ*fliY* Δ*fliN*/pDRH6461	This study
DRH6676	81-176 *rpsL*^{Sm} Δ*fliH fliY*::*kan-rpsL*	This study
DRH6713	81-176 *rpsL*^{Sm} Δ*fliY* Δ*fliH*	This study
DRH6723	81-176 *rpsL*^{Sm} Δ*fliY* Δ*fliH*/pDRH6472	This study
DRH6726	81-176 *rpsL*^{Sm} Δ*fliY* Δ*fliH*/pDRH6456	This study
DRH6856	81-176 *rpsL*^{Sm} Δ*fliY*/pDRH6461	This study
DRH6871	81-176 *rpsL*^{Sm} Δ*fliH fliN::kan*	This study
DRH7057	81-176 *rpsL*^{Sm} Δ*fliH fliN::kan*/pDRH6461	This study
DRH7060	81-176 *rpsL*^{Sm} Δ*fliH fliN::kan*/pDRH6456	This study
CRG479	81-176 *rpsL*^{Sm}/pDAR964	(7)
CRG1004	81-176 *rpsL*^{Sm} Δ*fliM*	This study
CRG1005	81-176 *rpsL*^{Sm} Δ*fliM*	This study
CRG1760	81-176 *rpsL*^{Sm} Δ*fliM fliN::cat-rpsL*	This study

JMB544	81-176 *rpsL*^{Sm} Δ*fliH*	This study
JMB1242	81-176 *rpsL*^{Sm} Δ*fliG*	(5)
JMB1903	81-176 *rpsL*^{Sm} Δ*astA* Δ*fliG*/pJMB1746	(5)
JMB2046	81-176 *rpsL*^{Sm} Δ*astA* Δ*fliF*/pJMB2032	(5)
SNJ311	81-176 *rpsL*^{Sm} *fliH*::*cat-rpsL*	This study
WPK611	81-176 *rpsL*^{Sm} *fliY*::*kan-rpsL*	This study
WPK633	81-176 *rpsL*^{Sm} *fliN*::*kan*	This study
WPK662	81-176 *rpsL*^{Sm} Δ*fliY*	This study
WPK663	81-176 *rpsL*^{Sm} Δ*fliY*	This study
WPK672	81-176 *rpsL*^{Sm} Δ*fliM* Δ*fliY*	This study
