# Supplementary material for: Diversification of Campylobacter jejuni Flagellar C-Ring Composition Impacts Its Structure and Function in Motility, Flagellar Assembly, and Cellular Processes
Source: mBio. 2020 Jan 7;11(1):e02286-19. doi: 10.1128/mBio.02286-19 (PMC6946799; doi:10.1128/mBio.02286-19)
Supplement: TABLE S2 [file mBio.02286-19-st002.pdf]

Table S2. Plasmids used in this study

Strain	Genotype	Source/Reference
pBR322	Amp^R	New England Biolabs
pUC19	Amp^R	New England Biolabs
pQE30	Amp^R; expression vector for addition of 6XHis tag to N-terminus of proteins	Qiagen
pGEX-4T-2	Amp^R; expression vector for addition of cytoplasmic glutathione S-transferase to the N-terminus of proteins	GE Healthcare
pMAL-c2X	Amp^R; expression vector for addition of cytoplasmic maltose-binding protein to the N-terminus of proteins	New England Biolabs
pDAR964	Cat^R; *E. coli-C. jejuni* shuttle vector containing *cat* promoter with an in-frame N-terminal FLAG sequence for expression of genes for complementation	(7)
pECO102	Cat^R; *E. coli-C. jejuni* shuttle vector containing *cat* promoter for expression of genes for complementation	(8)
pILL600	Source of the *Campylobacter aphA-3* (*kan*) cassette	(9)
pDRH265	pUC19 containing *cat-rpsL*	(1)
pDRH437	pUC19 containing *kan-rpsL*	(10)
pDRH1367	pUC19 containing *fliN*::*cat-rpsL*	(11)
pDRH1506	pUC19 containing *fliI*::*cat-rpsL*	(6)
pDRH6027	pJMB533 with SmaI-fragment containing *kan* cassette from pILL600 cloned into the EcoRV site in *fliN* in pJMB533	This study
pDRH6265	pUC19 with in-frame fusion of the start codon of *fliM* to the penultimate codon of *fliY* to delete the coding sequences of both *fliM* and *fliY* cloned into the PstI site	This study
pDRH6436	pECO102 with *fliM* with a C-terminal FLAG tag added to the penultimate codon of *fliM* cloned into the BamHI and PstI sites	This study
pDRH6456	pDAR964 with *fliH* coding sequence from codon 2 to the penultimate codon cloned into the BamHI and PstI sites to add a C-terminal FLAG-tag	This study
pDRH6458	pDAR964 with *fliI* coding sequence from codon 2 to the penultimate codon cloned into the BamHI and PstI sites to add a C-terminal FLAG-tag	This study
pDRH6461	pDAR964 with *fliN* coding sequence from codon 2 to the penultimate codon cloned into the	This study

	BamHI and PstI sites to add a C-terminal FLAG-tag	
pDRH6462	pQE30 with *fliH* coding sequence cloned in frame into the BamHI site	This study
pDRH6472	pDAR964 with *fliY* coding sequence from codon 2 to the penultimate codon cloned into the BamHI and PstI sites to add a C-terminal FLAG-tag	This study
pABT841	pUC19 containing *fliS*::*cat-rpsL*	(12)
pCRG1425	pJMB1971 with *SmaI*-fragment containing *kan-rpsL* inserted into SwaI site within *fliY*	This study
pJMB523	pUC19 with in-frame fusion of codons 43 to the stop codon of *fliH* to delete the last 233 amino acids cloned into the BamHI site	This study
pJMB533	pUC19::*fliN* locus	(5)
pJMB537	pUC19 containing *fliN*::*cat-rpsL*	(5)
pJMB572	pUC19 containing *fliM*::*cat-rpsL*	(11)
pJMB1249	pUC19 with in-frame deletion of codons 29 to 79 of *fliN* cloned into the BamHI site	This study
pJMB1401	pUC19::Δ*fliM*	(5)
pJMB1460	pQE30 with *fliN* coding sequence cloned in frame into the BamHI site	This study
pJMB1732	pMAL-c2X with *fliM* coding sequence cloned in-frame into the BamHI site	This study
pJMB1971	pBR322 with a mutation of bases $T_{132}A$ and $A_{133}T$ to create a SwaI site in *fliY*	(5)
pJMB2035	pUC19 with an in-frame deletion *fliY* by fusing the start and stop codons cloned into the BamHI site	This study
pMB120	pGEX-4T-2 with a *flhG* coding sequence clones in frame into the BamHI site	This study
pSNJ280	pUC19 with *fliH* locus cloned into the BamHI sites	This study
pSNJ281	pSNJ280 with StuI site created in *fliH* by changing nucleotides 184-186 of coding sequence from CAT to GGT	This study
pSNJ301	pSNJ281 with SmaI-fragment containing *cat-rpsL* inserted into the StuI site of *fliH*	This study
