# Supplementary material for: Diversification of Campylobacter jejuni Flagellar C-Ring Composition Impacts Its Structure and Function in Motility, Flagellar Assembly, and Cellular Processes
Source: mBio. 2020 Jan 7;11(1):e02286-19. doi: 10.1128/mBio.02286-19 (PMC6946799; doi:10.1128/mBio.02286-19)
Supplement: TEXT S1 [file mBio.02286-19-s0001.pdf]

Text S1. Additional Materials and Methods

Construction of mutants. *C. jejuni* mutants were constructed with plasmids purified from *E. coli* that were introduced into *C. jejuni* by electroporation or natural transformation after methylation modification with EcoRI methyltransferase following previously described methods (1, 2). Cloning of DNA into plasmids was accomplished by standard procedures using T4 DNA ligase or Gibson Assembly Mastermix (New England Biolabs). For cloning of genes to be deleted from the *C. jejuni* 81-176 *rpsL*^{Sm} (DRH212) chromosome, DNA fragments containing approximately 750 bases upstream and downstream of the target gene to be mutated was amplified by PCR using primers containing 5' BamHI restriction sites. For creation of DNA fragments with restriction sites within coding sequences of genes or in-frame deletions of specific genes, primers were designed to create two DNA fragments with the mutations, and then the mutations were joined by PCR. All fragments were then cloned into the BamHI site of pUC19. All DNA fragments cloned were verified by sequencing to result in: pSNJ280 (pUC19::*fliH*), pDRH6265 (pUC19::Δ*fliMY*), pJMB2035 (pUC19::Δ*fliY*), pSNJ281 which contains a StuI site within the coding sequence of *fliH* in pSNJ280, pJMB523 (pUC19::Δ*fliH*), and pJMB1249 (pUC19::Δ*fliN*).

A SmaI-digested *cat-rpsL* cassette (from pDRH265), *kan-rpsL* cassette (from pDRH437), or *kan* cassette (from pILL600) was ligated into plasmids in the appropriate restriction sites to interrupt each gene. Plasmids were introduced into *C. jejuni* 81-176 *rpsL*^{Sm} to interrupt genes on the chromosome. Mutants were recovered on MH agar containing chloramphenicol or kanamycin and verified by colony PCR and the following isogenic mutants of 81-176 *rpsL*^{Sm} were obtained: DRH3302 (*fliM*::*cat-rpsL*), SNJ311 (*fliH*::*cat-rpsL*), WPK611 (*fliY*::*kan-rpsL*), and WPK633 (*fliN*::*kan*). Plasmids containing in-frame deletion of genes were introduced into

strains containing respective *cat-rpsL* or *kan-rpsL* interruption of genes to replace the interrupted genes with the in-frame deletion mutants on the chromosome of *C. jejuni*. Transformants were recovered on MH agar containing a range of streptomycin concentrations and then screened for chloramphenicol or kanamycin sensitivity. Deletion of each gene was verified by colony PCR, which resulted in the creation of the following 81-176 *rpsL*^{Sm} mutant strains: CRG1004 and CRG1005 (two separate Δ*fliM* mutants), JMB544 (Δ*fliH*), WPK662 and WPK663 (two separate Δ*fliY* mutants), and WPK672 (81-176 *rpsL*^{Sm} Δ*fliM* Δ*fli*Y). Other double mutants were constructed similarly as described above by first introduction of a plasmid to interrupt a second gene with a *cat-rpsL* or *kan-rpsL* cassette in an in-frame deletion mutant to result in DRH6676 (Δ*fliH fliY*::*kan-rpsL*), DRH6871 (Δ*fliH fliN*::*kan*), CRG1760 (Δ*fliM fliN*::*cat-rpsL*), DRH6609 (Δ*fliY fliN*::*cat-rpsL*), DRH5332 (Δ*fliH fliI*::*cat-rpsL*), and DRH6411 (Δ*fliH fliS*::*cat-rpsL*). Appropriate plasmids were introduced into these strains to replace the interrupted gene on the chromosome with an in-frame deletion construct of the second targeted gene to result in the creation of DRH6630 (Δ*fliY* Δ*fliN*), DRH6713 (Δ*fliY* Δ*fliH*), and DRH6432 (Δ*fliM* Δ*fliN*).

Plasmids for complementation of Δ*fliY*, Δ*fliN*, Δ*fliH*, and Δ*fliI* mutants were constructed by amplifying from 81-176 chromosomal DNA the coding sequences of genes from the second codon through the stop codon with an in-frame BamHI or PstI sites added to the 5' end of the fragments. These fragments were cloned into BamHI- and PstI-digested pDAR964, which fused in frame a start codon and DNA sequence encoding a FLAG tag and a constitutive promoter from *cat*, encoding chloramphenicol acetyltransferase for expression to the 5' end of fragments. DNA sequencing verified the construction of the following plasmids: pDRH6456 (pDAR964::*fliH*), pDRH6458 (pDAR964::*fliI*), pDRH6461 (pDAR964::*fliN*), and pDRH6472 (pDAR964::*fliY*). Plasmids were transferred to DH5α/pRK212.1 for conjugation into appropriate

C. jejuni 81-176 *rpsL*^{Sm} mutants. Due to toxicity of expressing *C. jejuni fliM* in *E. coli* from the constitutive *cat* promoter, *fliM* was amplified from the 81-176 chromosome with primers designed to add 5' BamHI site fused in-frame to codon 2 and sequence for a FLAG tag, stop codon and PstI site fused in-frame to the penultimate codon. This DNA fragment was then cloned into pECO102 that was purified from *C. jejuni* 81-176 and digested with BamHI and PstI and transformed directly into CRG1004 or CRG1005 (81-176 *rpsL*^{Sm} Δ*fliM*) to create pDRH6436.

Antibody generation. Recombinant *C. jejuni* proteins were induced from expression plasmids for purification and antibody generation. The coding sequences for *flhG*, *fliM*, *fliN*, and *fliH* were amplified form the *C. jejuni* 81-176 genome using primers that fused in-frame BamHI sites to the start and stop codons of the genes. These PCR fragments were digested with BamHI and then ligated into BamHI-digested pGEX-4T-2 (for *flhG*), pMAL-c2X (for *fliM*) and pQE30 (for *fliN* and *fliH*) to create pMB120, pJMB1732, pJMB1460, and pDRH6462, respectively. After cloning, all genes were sequenced for correct construction.

pMB120 was transformed into BL21(DE3) for induction and purification of GST-FlhG. An overnight culture was diluted 1:40 in 1 L LB with ampicillin and grown at 37 °C to an OD_{600} 0.5. Production of recombinant protein was induced with 0.1 mM IPTG and the culture was incubated for an additional 4 h. Bacteria were passaged through an EmulsiFlex-C5 disrupter three times at 15,000 – 20,000 lb/in^2. The soluble fractions were recovered by centrifugation at 13,000 rpm for 2 h. GST-FlhG was purified using Glutathione Sepharose 4B and FlhG was released from with 10 mM glutathione according to manufacturer's instructions (GE Healthcare).

pJMB1460 was transformed into XLI-Blue for induction and purification of 6XHis-FliN. An overnight culture was diluted 1:40 in 2 L LB with ampicillin and grown at 37 °C to an OD_{600}

0.5. Production of recombinant protein was induced with 1 mM IPTG and the culture was incubated for an additional 4 h. The cell pellet was recovered by centrifugation at 6000 rpm for 10 min. The pellet was dissolved in 8 M urea, 0.5 M sucrose, and 1 mg/ml Zwittergen. Insoluble material was removed by centrifugation at 13,000 for 30 min. The recovered soluble fraction was further disrupted by an Emulsiflex-C5 disrupter three times at 15,000 – 20,000 lb/in^2 and the soluble fraction was recovered by centrifugation at 13,000 rpm for 2 h. Recombinant 6XHis-FliH and 6XHis-FliN proteins were purified using Ni-Nta beads according to manufacturer's instructions (Qiagen).

pJMB1732 was transformed into BL21(DE3) for induction and purification of MBP-FliM. An overnight culture was diluted 1:40 in 2 L LB with ampicillin and grown at 37 °C to an OD_{600} 0.5. Production of recombinant protein was induced with 0.25 mM IPTG and the culture was incubated for an additional 4 h. Bacteria were passaged through an EmulsiFlex-C5 disrupter three times at 15,000 – 20,000 lb/in^2. The soluble fractions were recovered by centrifugation at 13,000 rpm for 2 h. MBP-FliM was purified using amylose resin and FliM was released from MBP and resin by on-column digestion with 1:100 dilution of factor Xa at 3 h at 4 °C according to manufacturer's instructions (New England Biolab).

Purified FliM was used to immunize mice and purified GST-FlhG, 6XHis-FliH and 6XHis-FliN were used to immunize guinea pigs by standard procedures for antisera generation via a commercial vendor (Cocalico Biologicals). All use of animals in experimentation has been approved by IACUC at the University of Texas Southwestern Medical Center.

References

1. **Hendrixson DR, Akerley BJ, DiRita VJ.** 2001. Transposon mutagenesis of *Campylobacter jejuni* identifies a bipartite energy taxis system required for motility. Mol Microbiol **40:**214-224.
2. **Beauchamp JM, Leveque RM, Dawid S, DiRita VJ.** 2017. Methylation-dependent DNA discrimination in natural transformation of *Campylobacter jejuni*. Proc Natl Acad Sci U S A **114:**E8053-E8061.
3. **Figurski DH, Helinski DR.** 1979. Replication of an origin-containing derivative of plasmid RK2 dependent on a plasmid function provided in trans. Proc Natl Acad Sci U S A **76:**1648-1652.
4. **Bullock WO, Femandex JM, Short JM.** 1987. XL1-Blue: a high efficiency plasmid transforming *recA Escherichia coli* strain with beta-galactosidase selection. Biotechniques **5:**376-378.
5. **Boll JM, Hendrixson DR.** 2013. A regulatory checkpoint during flagellar biogenesis in *Campylobacter jejuni* initiates signal transduction to activate transcription of flagellar genes. mBio **4:**e00432-13.
6. **Joslin SN, Hendrixson DR.** 2009. Activation of the *Campylobacter jejuni* FlgSR two-component system is linked to the flagellar export apparatus. J Bacteriol **191:**2656-2667.
7. **Gulbronson CJ, Ribardo DA, Balaban M, Knauer C, Bange G, Hendrixson DR.** 2016. FlhG employs diverse intrinsic domains and influences FlhF GTPase activity to numerically regulate polar flagellar biogenesis in *Campylobacter jejuni*. Mol Microbiol **99:**291-306.

8. **Wiesner RS, Hendrixson DR, DiRita VJ.** 2003. Natural transformation of *Campylobacter jejuni* requires components of a type II secretion system. J Bacteriol **185:**5408-5418.

9. **Labigne-Roussel A, Harel J, Tompkins L.** 1987. Gene transfer from *Escherichia coli* to *Campylobacter* species: development of shuttle vectors for genetic analysis of *Campylobacter jejuni*. J Bacteriol **169:**5320-5323.

10. **Hendrixson DR, DiRita VJ.** 2003. Transcription of σ^{54}-dependent but not σ^{28}-dependent flagellar genes in *Campylobacter jejuni* is associated with formation of the flagellar secretory apparatus. Mol Microbiol **50:**687-702.

11. **Balaban M, Hendrixson DR.** 2011. Polar flagellar biosynthesis and a regulator of flagellar number influence spatial parameters of cell division in *Campylobacter jejuni*. PLoS Pathog **7:**e1002420.

12. **Barrero-Tobon AM, Hendrixson DR.** 2014. Flagellar biosynthesis exerts temporal regulation of secretion of specific *Campylobacter jejuni* colonization and virulence determinants. Mol Microbiol **93:**957-974.
